# Achieving sustainable goals in agroecosystems through the optimization of agricultural resources integrating the water-nitrogen-carbon nexus

**DOI:** 10.1016/j.isci.2025.112101

**Published:** 2025-02-25

**Authors:** Mo Li, Pingan Zhang, Aizheng Yang, Xiaofang Wang, Yan Sha, Qiang Fu

**Affiliations:** 1 School of Water Conservancy and Civil Engineering, Northeast Agricultural University, Harbin, Heilongjiang 150030, China; 2 Heilongjiang Province Key Laboratory of Smart Water Network, Northeast Agricultural University, Harbin, Heilongjaing 150030, China; 3 National Key Laboratory of Smart Farm Technology and System, Harbin, Heilongjiang 150030, China; 4 International Cooperation Joint Laboratory of Health in Cold Region Black Soil Habitat of the Ministry of Education, Harbin, Heilongjiang 150030, China; 5 Key Laboratory of Effective Utilization of Agricultural Water Resources of Ministry of Agriculture, Northeast Agricultural University, Harbin, Heilongjiang 150030, China

**Keywords:** environmental science, natural resources, bioresources, agricultural science, agricultural research and development

## Abstract

This study investigates the synergistic utilization of straw, water, and nitrogen resources in agricultural ecosystems to optimize sustainable management practices. Focusing on a tomato-corn-soybean agroecosystem, the research quantifies the interactions within the water-nitrogen-carbon nexus and evaluates various straw utilization strategies. Results show that, under optimized water (196-157-123 mm) and nitrogen (207-236-84 kg/ha) conditions, the water and carbon footprints decreased by 2.25–5.46% and 3.37–13.82%, respectively, compared to local practices. Economic benefits and overall quality improved by 8.27–21.06% and 4.06–7.63%, respectively. Prioritizing straw return to the field and straw biochar preparation scenarios enhanced system coordination by 1.41–9.62%. These findings offer valuable insights for decision-makers, providing strategies for sustainable agriculture that balance resource optimization, food security, and environmental protection.

## Highlights

• Developed an optimal framework for managing water, nitrogen, and carbon
• Proposed a recycling strategy for resources to achieve multi-dimensional goal synergy
• Addressed complexity and uncertainty in straw recycling for improved resource management
• Enhanced productivity and reduced emissions through straw return and biochar preparation

## Graphical abstract

**Figure:**
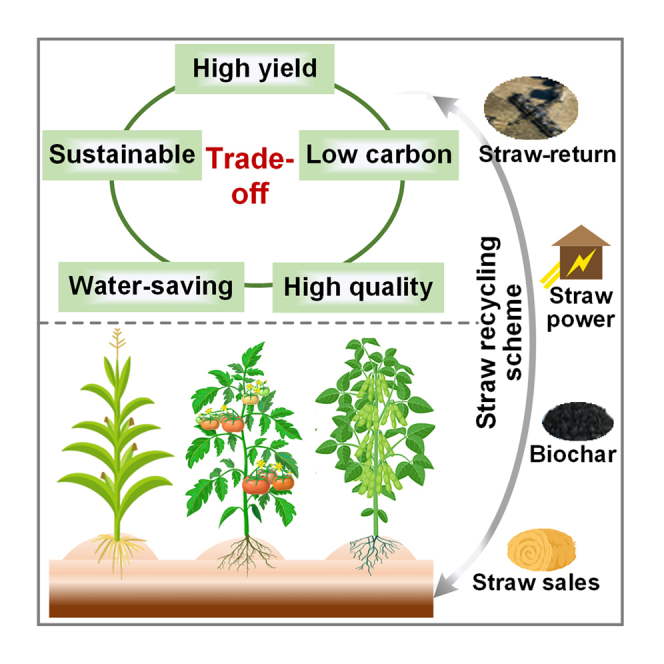

## Introduction

Population growth, water scarcity, the energy crisis, and climate change pose serious challenges to agricultural production.^1^ Agriculture is not only a key area for food production and economic growth but also plays an important role in greenhouse gas (GHG) emissions and waste management, contributing 13% and 40% of global GHG emissions and waste, respectively.^2^ To address these challenges, there is an urgent need to shift to diversified, sustainable agricultural production models that ensure food security while reducing negative environmental impacts (Hu et al., 2017). The traditional agricultural model relies heavily on resources such as water and nitrogen, ignoring the potential utilization of straw waste, which restricts the sustainable development of farmland ecosystems.^3^ Therefore, it is important to explore innovative agroecological models based on resource recycling to improve crop yields, enhance quality, improve resource use efficiency, and reduce environmental pollution, thereby achieving the sustainable development goals of agriculture.

Research on the relationships among water, nitrogen, and carbon has become an important way to promote the green and efficient use of agricultural resources and solve the problem of sustainable agricultural development.^4^ The interaction of water, nitrogen, carbon, and other resources in farmland ecosystems directly affects agricultural production and environmental health, and the management of the water-nitrogen-carbon relationship has played an increasingly critical role in balancing multidimensional goals such as those related to the economy, resources, and environment.^5^^,^^6^ Scholars have explored the interaction between the water-nitrogen-carbon relationship and economic-resource-environmental goals at regional scales, particularly by assessing the impact of water and fertilizer management strategies on models, such as farmland outputs, resource inputs, and footprints. While ensuring crop yields, optimizing crop quality for economic efficiency, resource sustainability, and environmental friendliness has become a key task.^7^ In particular, in the context of climate change, optimization models provide important assessment tools for decision-makers to assess the efficiency and sustainability of farmland ecosystems comprehensively, accounting for factors such as water and fertilizer management, crop planting structure, and climatic conditions.^8^ The relationships among the economy, resources, and environment could be better balanced by optimizing the model, leading to the achievement of sustainable management of agricultural resources.^9^ However, the application of optimization models often faces the challenges of data uncertainty and complexity, and most existing studies focus on the regulation of water and nitrogen resources at the farmland scale and lack comprehensive consideration of factors, such as crop quality, climate change, and straw use.^10^ Therefore, the combination of field experiments and multidimensional target optimization models is helpful for achieving the coordinated development of the goals of grain yield improvement, crop quality improvement, and environmental pollution reduction.

Circular agriculture provides a feasible path for the green and efficient use of agricultural resources, which could improve economic, resource, and environmental benefits and promote the sustainable management of farmland ecosystems.^11^ Based on the principle of multilevel recycling of resources, the model emphasizes ecosystem protection and sustainable use and aims to reduce external resource inputs, reduce agricultural biomass waste, improve resource recycling efficiency, and reduce GHG emissions.^12^ In agricultural production, straw, as the main waste, is often regarded as garbage or directly incinerated, resulting in waste of resources and environmental pollution.^13^ However, with circular agriculture models, straw waste can be converted into biomass energy or organic fertilizer to improve soil quality.^14^ The diversified use of straw could not only improve the soil structure and water retention capacity but also fix and recycle nutrients, such as nitrogen and carbon, reduce fertilizer dependence, and improve agroecosystem productivity.^15^ The recycling of straw is closely related to the water-nitrogen-carbon relationship, which can significantly improve resource use efficiency and reduce environmental pollution.^16^ However, most of the current studies focused on water and nitrogen management at the farmland scale, and there is a lack of comprehensive discussion on the diversified use of straw.^17^ Therefore, incorporating the comprehensive utilization of straw resources into the optimization of the water-nitrogen-carbon relationship would help to realize the efficient utilization of resources and promote the development of agriculture in a green and sustainable direction.

**Figure 1:**
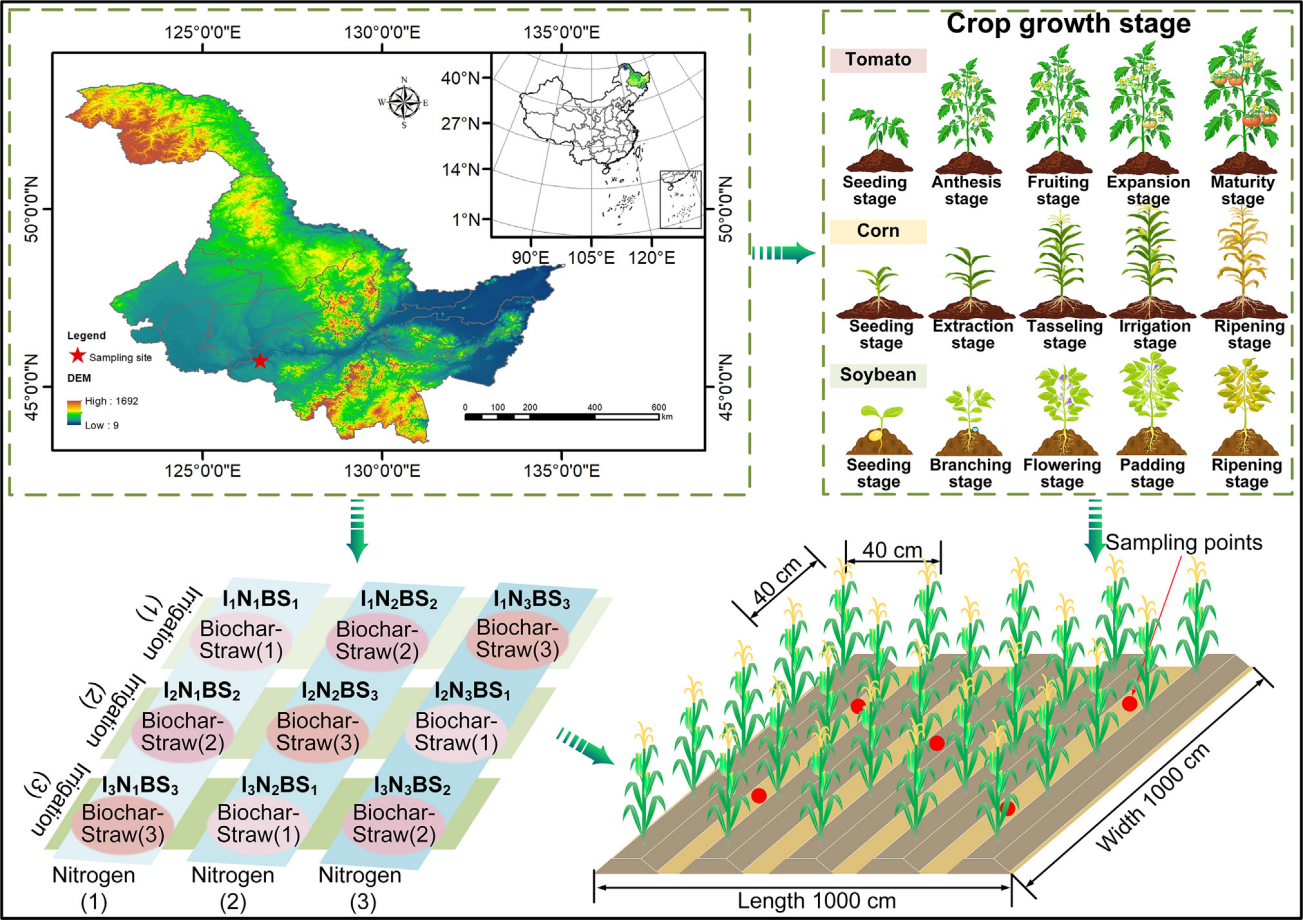
Experimental layout of the farmland community

**Figure 2:**
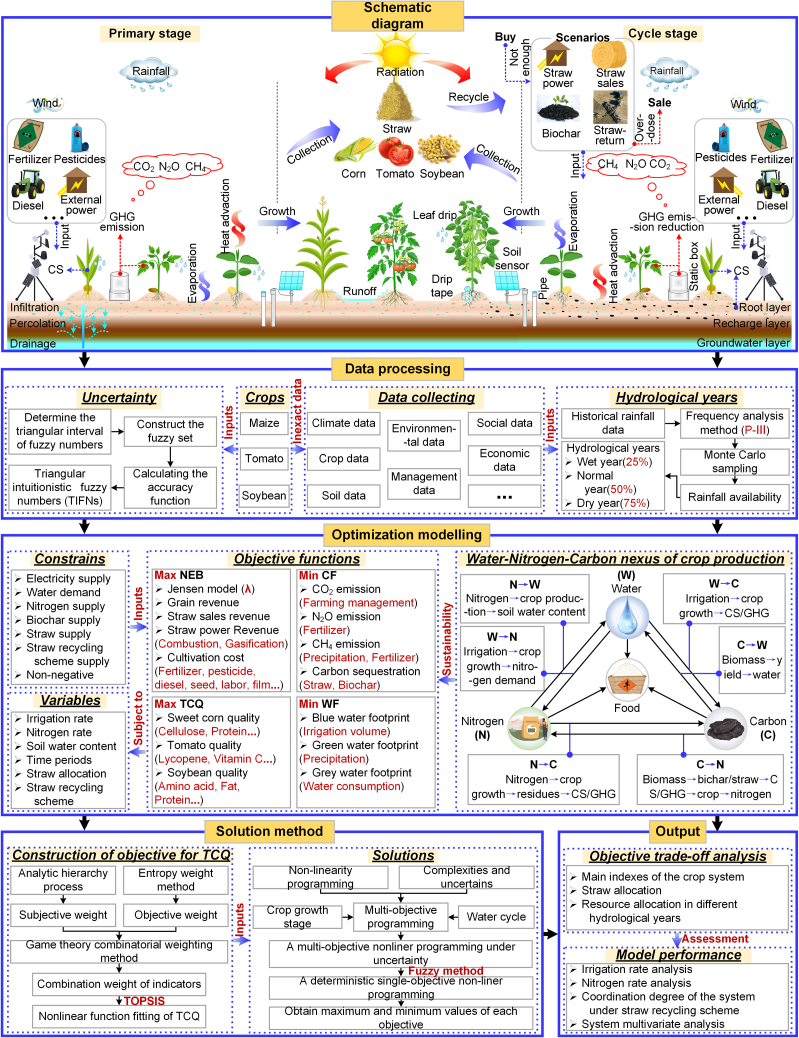
Model decision framework

**Figure 3:**
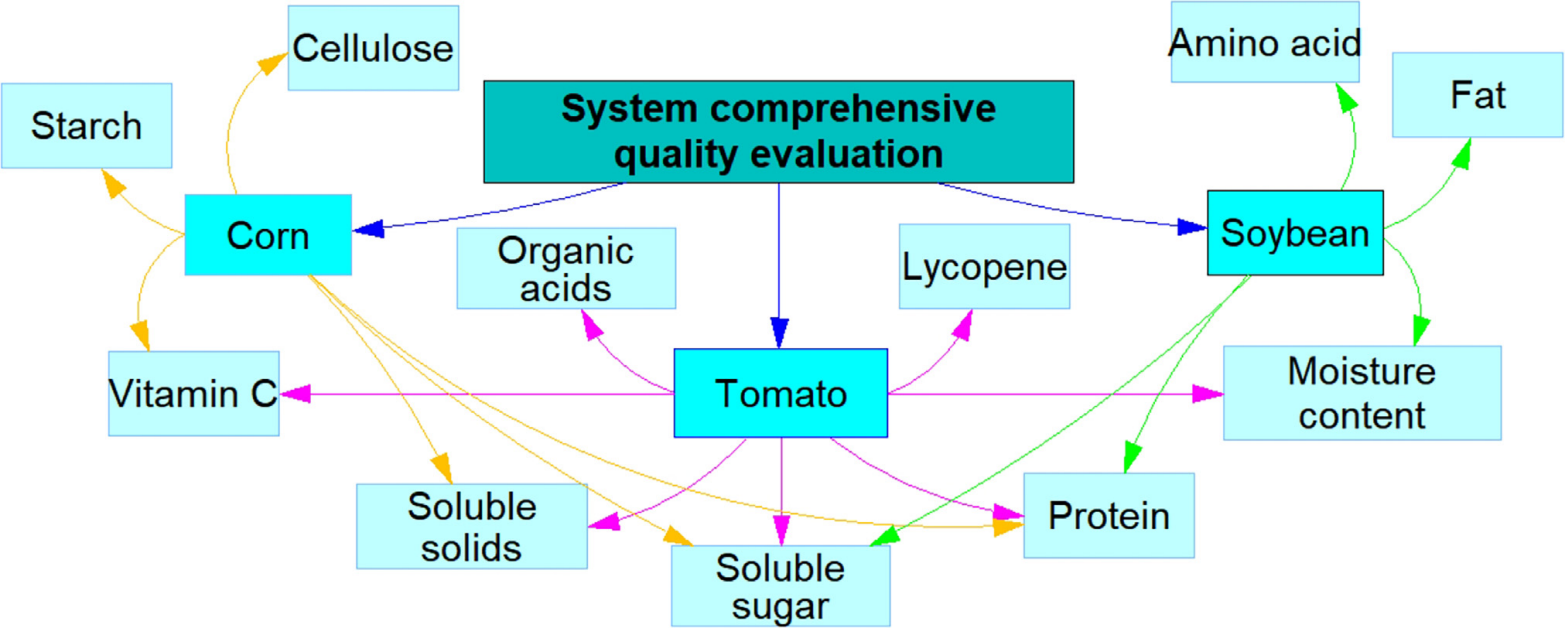
Framework of integrated crop quality in agroecosystems

Therefore, this study focused on the farmland ecosystem of tomato-maize-soybean crops (Figure 1), with important food and economic value and aimed to achieve green and efficient regulation of water, nitrogen, and straw resources through water-nitrogen-carbon relationship management. First, according to the principles of reduction, recycling, and reuse, mixed straw was systematically collected for crushing and returning to the field, biochar preparation, power generation, and sales (Figure 2). Second, considering the interaction between straw use and the combined application of water and nitrogen in farmland ecosystems, the net economic benefit (NEB), total comprehensive quality (TCQ) (Figure 3), water footprint (WF), and carbon footprint (CF) were incorporated into the multiobjective stochastic programming model, and an optimization framework for the sustainable management of agricultural resources oriented toward high yield, high quality, water savings, low carbon, and sustainability was constructed. Finally, the feasibility and applicability of the optimized model framework were verified in a tomato-maize-soybean farmland ecosystem. The optimization results provide a theoretical basis for the development of efficient and environmentally friendly circular agriculture and provide decision-making support for promoting the recycling of straw resources, the optimal allocation of water and nitrogen resources, and the protection of agricultural ecology.

## Results

### Optimizing model performance in resource regulation

**Figure 4:**
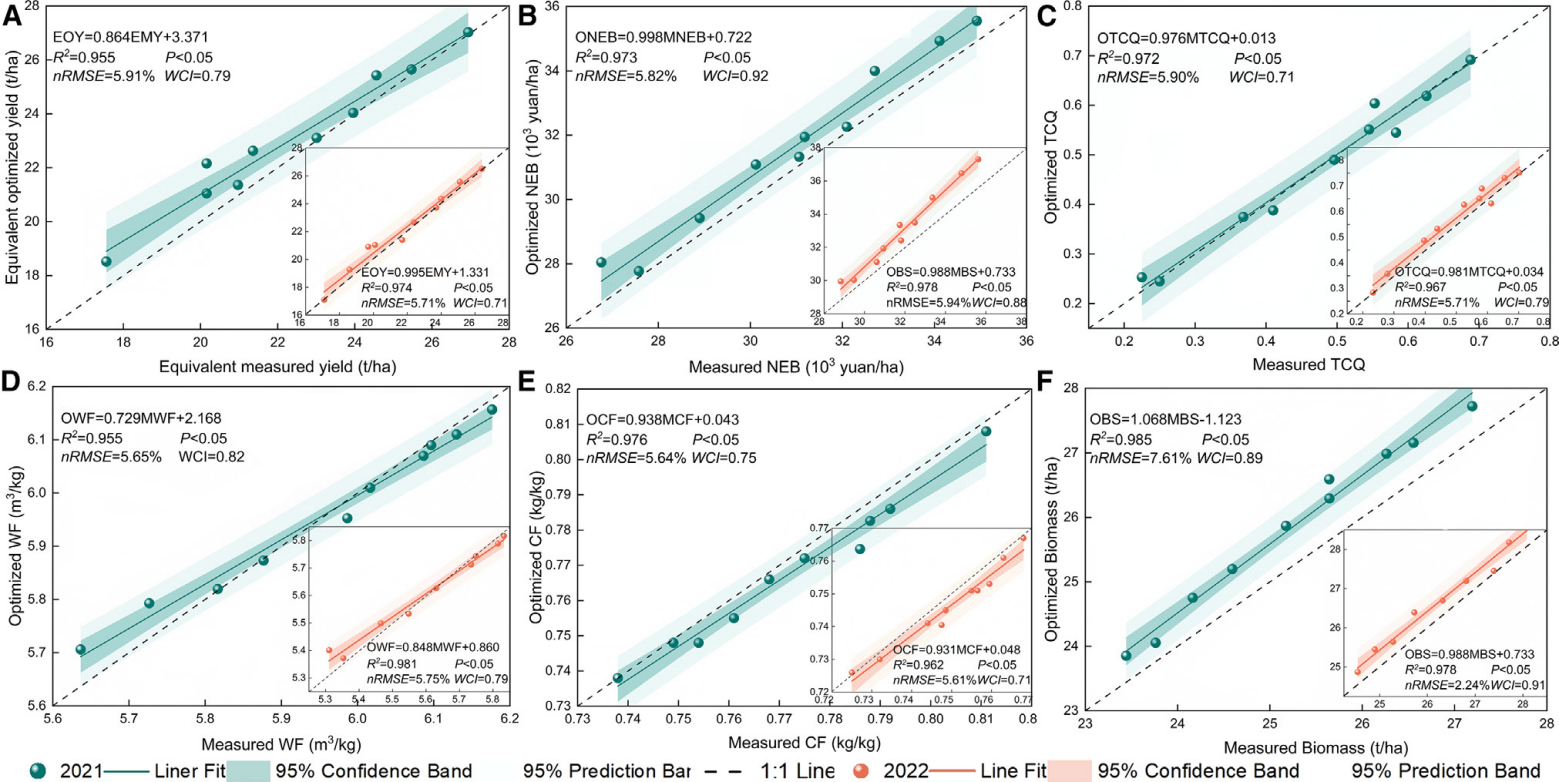
Comparison of simulated and measured values of yield (A), NEB (B), TCQ (C), WF (D), CF (E) and biomass (F)

The tomato, corn and soybean crops in the agroecosystem are from different genera, so the yield of each crop was converted to the “equivalent” yield of tomato for comparison. The values of the crop water sensitivity parameters at the beginning of the cycle (Tables 1, 2, and 3) revealed that the yield of tomato was strongly influenced by the fruiting and flowering stages; the yield of corn was strongly influenced by the tasseling and grubbing stages; and the yield of soybean was most sensitive to water at the flowering stage, followed by the meristemming and pod setting stages. When optimizing irrigation, the water supply at these fertility stages should meet crop growth demands as much as possible. Figure 4 shows the simulated yield, NEB, TCQ, WF, CF, and biomass values calculated on the basis of the constructed multiobjective optimization and regulation model with straw and biochar returned to the field and the fitting of the data to the measured values. As shown in Figure 4, the simulated values of yield, NEB, TCQ, WF, CF, and biomass were significantly correlated with the measured values (*R*^*2*^: 0.955–0.985; *nRMSE*: 2.24–5.94%; *WCI:* 0.71–0.92; *p* < 0.05) and had similar trends. Most of the points were concentrated within the 95% confidence interval, whereas a few points with higher deviations were concentrated within the 95% prediction interval. Among them, the simulated NEB values had higher *R*^*2*^ and *WCI* values and lower nRMSE values (*R*^*2*^: 0.973–0.978; *WCI*: 0.88–0.92; *nRMSE*: 5.82–5.94%; *p* < 0.05; Figure 4B). The differences between the simulated and measured values of biomass were relatively small (*R*^*2*^ = 0.978–0.985; *WCI* = 0.89–0.91; *nRMSE* = 2.24–7.61%, Figure 4F). Therefore, the synergistic multidimensional agroecosystem regulation model can accurately optimize yield, NEB, TCQ, WF, CF, and biomass under different treatments.

**Table 1:** Experimental design scheme

Year	2021/2022	2021/2022	2022
No	Irrigation amount (mm)	Nitrogen rate (kg/ha)	Additive rate (t/ha)
T1	*I*_*1*_	*N*_*1*_	*B*_*3*_+*S*_*3*_
T2	*I*_*1*_	*N*_*2*_	*B*_*1*_+*S*_*1*_
T3	*I*_*1*_	*N*_*3*_	*B*_*2*_+*S*_*2*_
T4	*I*_*2*_	*N*_*1*_	*B*_*2*_+*S*_*2*_
T5	*I*_*2*_	*N*_*2*_	*B*_*3*_+*S*_*3*_
T6	*I*_*2*_	*N*_*3*_	*B*_*1*_+*S*_*1*_
T7	*I*_*3*_	*N*_*1*_	*B*_*1*_+*S*_*1*_
T8	*I*_*3*_	*N*_*2*_	*B*_*2*_+*S*_*2*_
T9	*I*_*3*_	*N*_*3*_	*B*_*3*_+*S*_*3*_

**Table 2:** Weighting of straw allocation options under different scenarios

Scenarios	Weights			
	*Pr*_*straw*_	*Pr*_*biochar*_	*Pr*_*ele*_	*Pr*_*sell*_
*S*_*w1*_*: Pr*_*straw*_*=Pr*_*biochar*_*=Pr*_*ele*_*=Pr*_*sell*_	0.25	0.25	0.25	0.25
*S*_*w2*_*: Pr*_*straw*_*>Pr*_*biochar*_*>Pr*_*ele*_*>Pr*_*sell*_	0.55	0.20	0.15	0.10
*S*_*w3*_*: Pr*_*biochar*_*>Pr*_*ele*_*>Pr*_*sell*_*>Pr*_*straw*_	0.05	0.60	0.20	0.15
*S*_*w4*_*: Pr*_*ele*_*>Pr*_*sell*_*>Pr*_*straw*_*>Pr*_*biochar*_	0.14	0.06	0.50	0.30
*S*_*w5*_*: Pr*_*sell*_*>Pr*_*straw*_*>Pr*_*biochar*_*>Pr*_*ele*_	0.26	0.20	0.14	0.40

**Table 3:** Water sensitivity parameters for each fertility stage of crops at the beginning of system operation

Growth stage	Ⅰ^a^	Ⅱ^b^	Ⅲ^c^	Ⅳ^d^	Ⅴ^e^
Tomato	0.086	0.314	0.532	0.208	0.163
Corn	0.101	0.225	0.447	0.621	0.307
Soybean	0.018	0.685	0.812	0.416	0.124

a, b, c, d and e denote the seedling-seedling-seedling, anthesis-extraction-branching, fruiting-tasseling-flowering, expansion-irrigation-podding, and maturity-ripening-ripening stages, respectively, of the tomato-corn-soybean agroecosystem.

### Optimal allocation of water and nitrogen resources under different hydrological year scenarios

**Figure 5:**
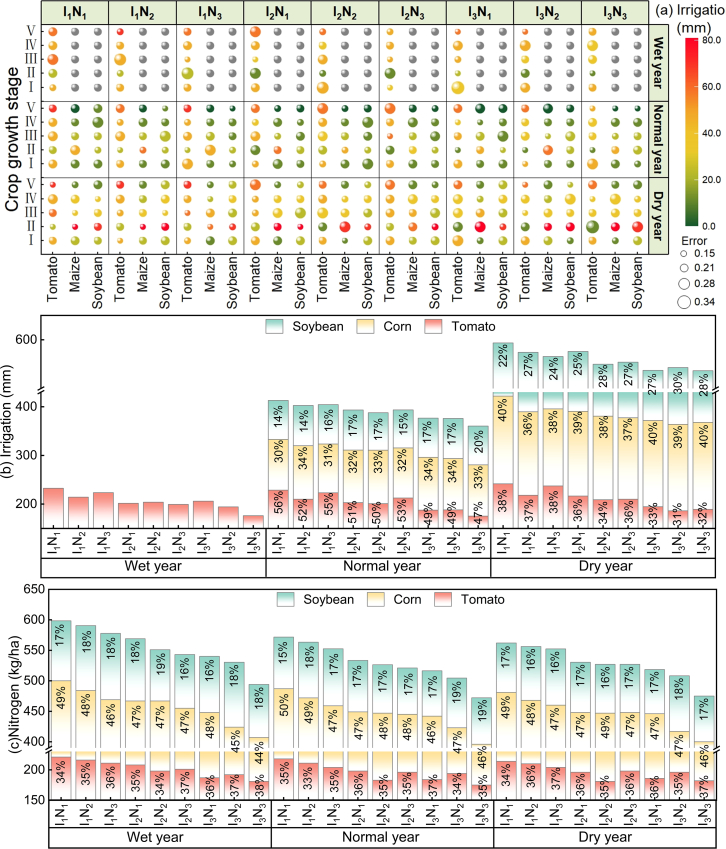
Optimized allocation of water and nitrogen resources in different hydrological years

The results of optimal water and nitrogen resource allocation in farmland ecosystems under different hydrological year scenarios were obtained by solving the established optimization model. Figure 5 shows not only the amount of irrigation water for crops at different fertility stages but also the contributions of the water and nitrogen resources of each crop in the agroecosystem under different hydrological year scenarios. As shown in Figure 5A, the tomato irrigation volumes of the different treatments were not significantly different (*p* > 0.05) under the different hydrological year scenarios and were greatest during the ripening period, which was attributed to the fact that the internal environment of the greenhouses was precisely controlled by artificial means and that the ripening period was determined based on the picking date of the first ear or fruit. Rainfall in the water-abundant year met the maximum water demand of corn and soybean, irrigation in the flat water year accounted for 14.2–22.9% and 9.8–16.6% of the total water consumption of corn and soybean, respectively, and irrigation in the dry water year accounted for 24.6–37.8% and 21.3–33.4% of the total water consumption of corn and soybean, respectively. Corn and soybean had the maximum irrigation at the elongation and extraction stages. This was due to the increase in rainfall during the reproductive period of the crops, which led to a decrease in the crops’ need for irrigation. As shown in Figure 5B, the amount of water irrigated in the agroecosystem in the high-water year was due to the greenhouse tomatoes, whereas corn and soybeans did not need to be irrigated. However, rainfall decreased significantly from the water-abundant year to the flat water year, and irrigation was needed for corn and soybeans to ensure crop production. When rainfall declined from the flat water year to the dry water year, irrigation needed to be increased to avoid drought-induced reductions in corn and soybean production. There was no significant difference (*p* > 0.05) in the amount of water irrigated for tomato in different hydrological years, and the amount of water irrigated for corn and soybeans in dry years significantly (*p* < 0.05) increased by 13.42–20.27% and 11.27–19.68%, respectively, compared with that in flat water years. As shown in Figure 5C, there was no significant difference in the amount of N applied to tomato in different hydrological years, which varied between 175 and 221 kg/ha (*p* > 0.05). Compared with the other treatments, the I1N1 treatment resulted in an average increase of 4.62–9.83%. The amount of nitrogen applied to corn and soybean varied significantly (*p* < 0.05) among the hydrological years, with average increases of 3.16–5.29% (1.05–3.15%) and 3.56–6.67% (2.16–4.66%) in the abundant water years compared with the flat water (dry water) years. The results showed that (1) varying the optimal allocation of water and nitrogen resources in different hydrological years is an effective measure for addressing extreme climate change and (2) the adoption of a reasonable water resource optimization scheme can effectively mitigate the impacts of rainfall on crop production during the critical fertility period of crops.

**Figure 6:**
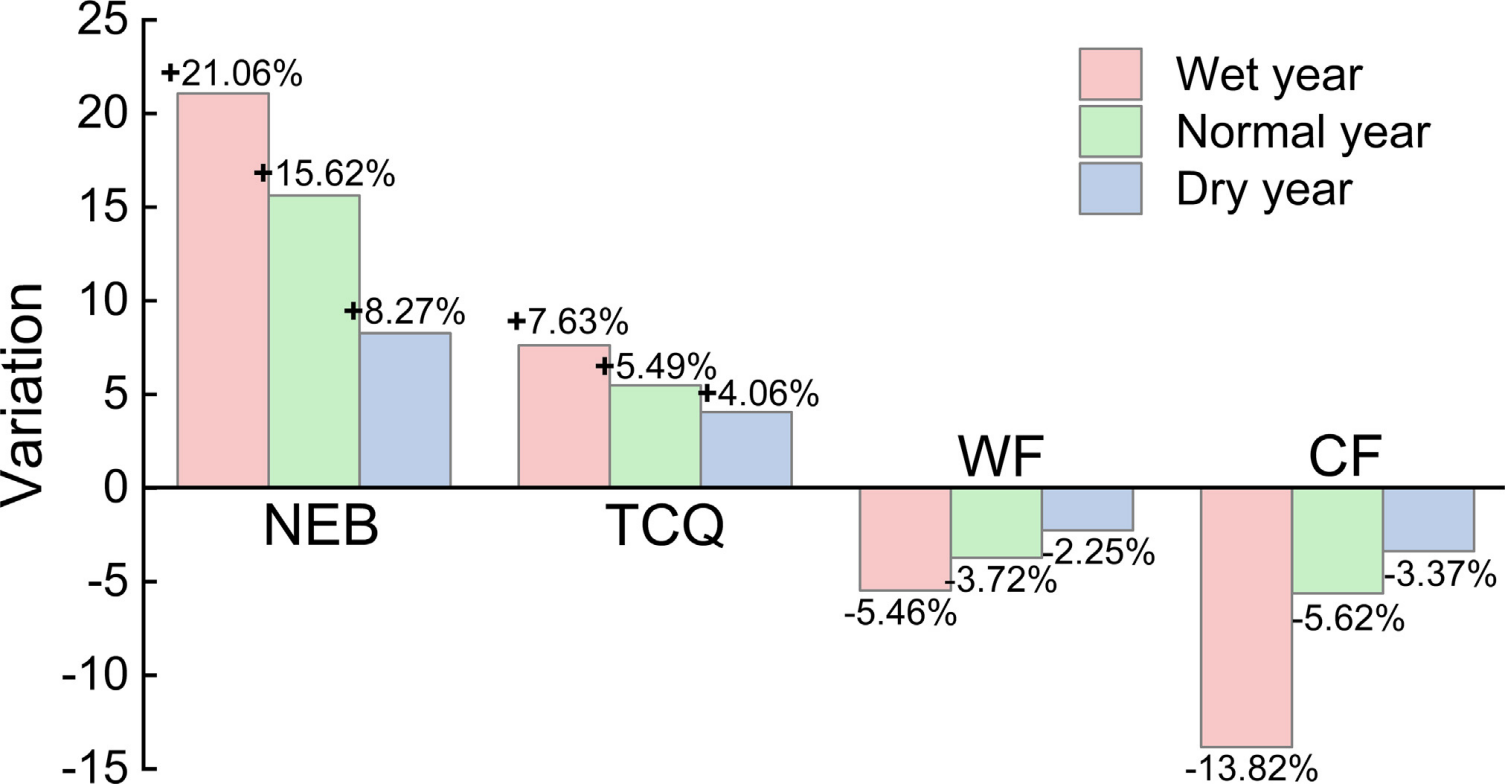
Comparison of the optimal results with the current state of research

In addition, significant differences (*p* < 0.05) between the optimal values of NEB, TCQ, WF, and CF (i.e., the optimal values considering only the corresponding single objective) and the status quo values of local farmers for different hydrological year scenarios were observed (Figure 6). When the water nitrogen use in the tomato-corn-soybean system was 196-157-123 mm and 207-236-84 kg/ha, respectively, the economic efficiency and overall quality increased by 8.27–21.06% and 4.06–7.63%, respectively, compared with the status quo. In addition, through the optimal allocation of water and nitrogen resources in farmland ecosystems, farmland GHG emissions can be effectively reduced while improving the efficiency of irrigation water use efficiency. In particular, the crop WF was reduced by 2.25–5.46%, and the CF was reduced by 3.37–13.82%. The farmland ecosystem optimization model was used to establish a rational management scheme for farmland water and nitrogen resources from the perspective of high efficiency and sustainability by taking crop yield as the central factor.

### Effects of straw allocation options on the optimization results

**Figure 7:**
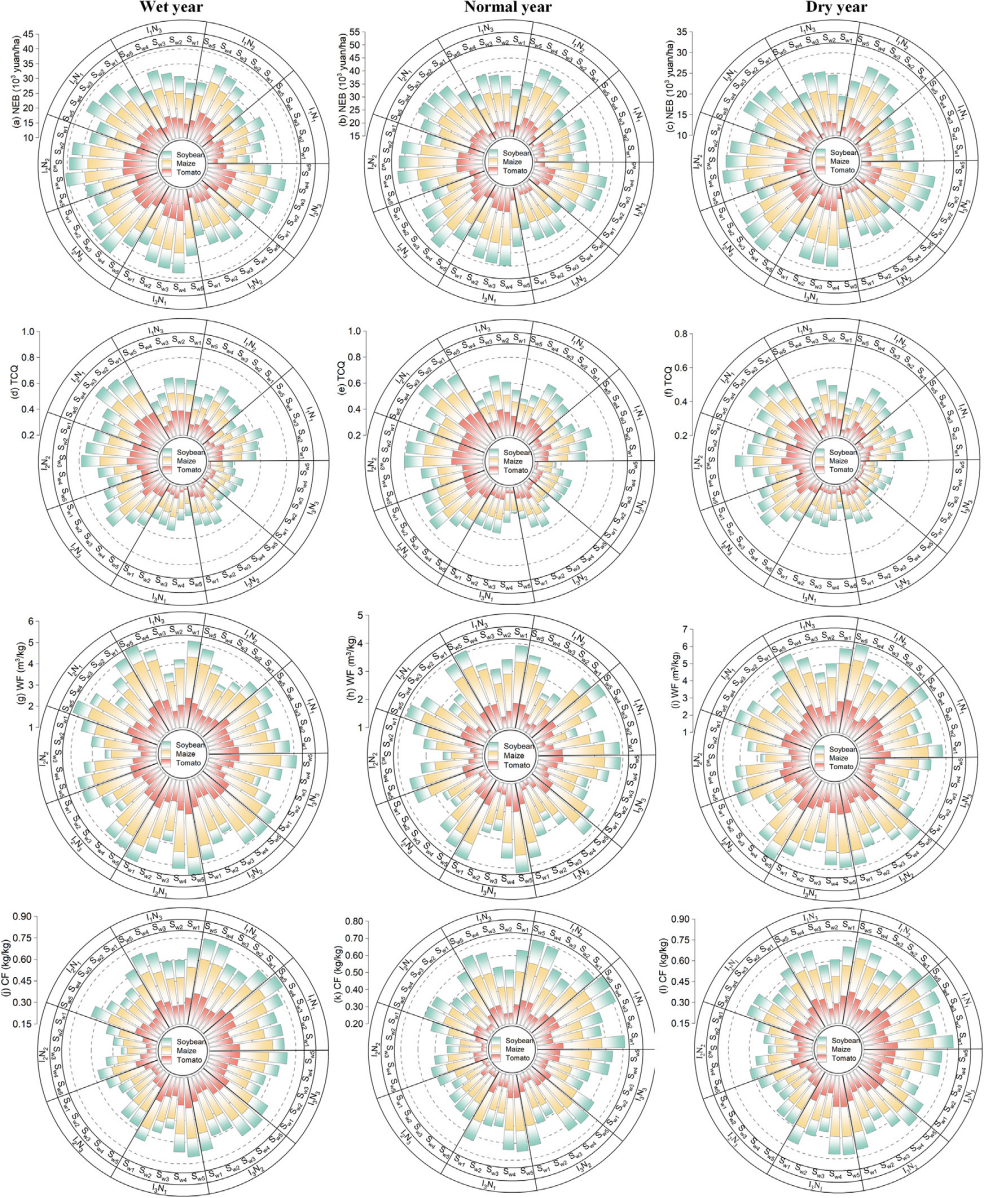
Effects of the straw allocation scheme on NEB, TCQ, WF, and CF

Different straw utilization schemes affect the optimal allocation of water and nitrogen resources in agroecosystems. Figure 7 shows the variations in NEB, TCQ, WF, and CF for each treatment when considering the weights of straw utilization schemes in different hydrological years. In particular, the detailed results of water and nitrogen resource allocation for each treatment under the different straw allocation schemes (see Figure S1). As shown in Figure 7, NEB varied from 13.5 to 52.6×10^3^ yuan/ha, TCQ varied from 0.21 to 0.89, WF varied from 3.6 to 6.4 m^3^/kg, and CF varied from 0.54 to 0.87 kg/kg. Taking the NEB of different hydrological years as an example, the NEB of each treatment was lower than the best optimized value in all the scenarios. When the straw power generation scenario was prioritized (S_w4_), the treatment optimization value of I_2_N_2_ in the abundant water year was closest to the best optimization value; when the straw sale scenario was prioritized (S_w5_), the treatment optimization value of I_3_N_2_ in the dry water year was farthest from the best optimization value. Moreover, the results show a conflict between the straw power generation and straw sale scenarios. When equal weights were given to the straw allocation schemes (S_w1_), the optimized value of the I_2_N_2_ treatment in flat water years was closest to the best optimized value. However, in special cases, i.e., when straw return (S_w2_) and biochar preparation (S_w3_) were prioritized, the NEB values of the two scenarios were similar, which was attributed to the similar weights of the scenarios, and there was no significant difference between hydrological years (*p* > 0.05). The results suggest that straw utilization schemes may compete or conflict under different hydrological year conditions and can be used to identify synergies and trade-offs in the model. TCQ had a similar pattern to that of NEB under the different straw allocation schemes, whereas WF and CF had patterns opposite to that of NEB. In addition, tomato made the greatest contribution to all straw allocation schemes, followed by corn and soybean. This may be because crops in agroecosystems come from different genera and families, which leads to differences in water and nitrogen allocation in the model, which in turn affects the contribution of the system.

**Figure 8:**
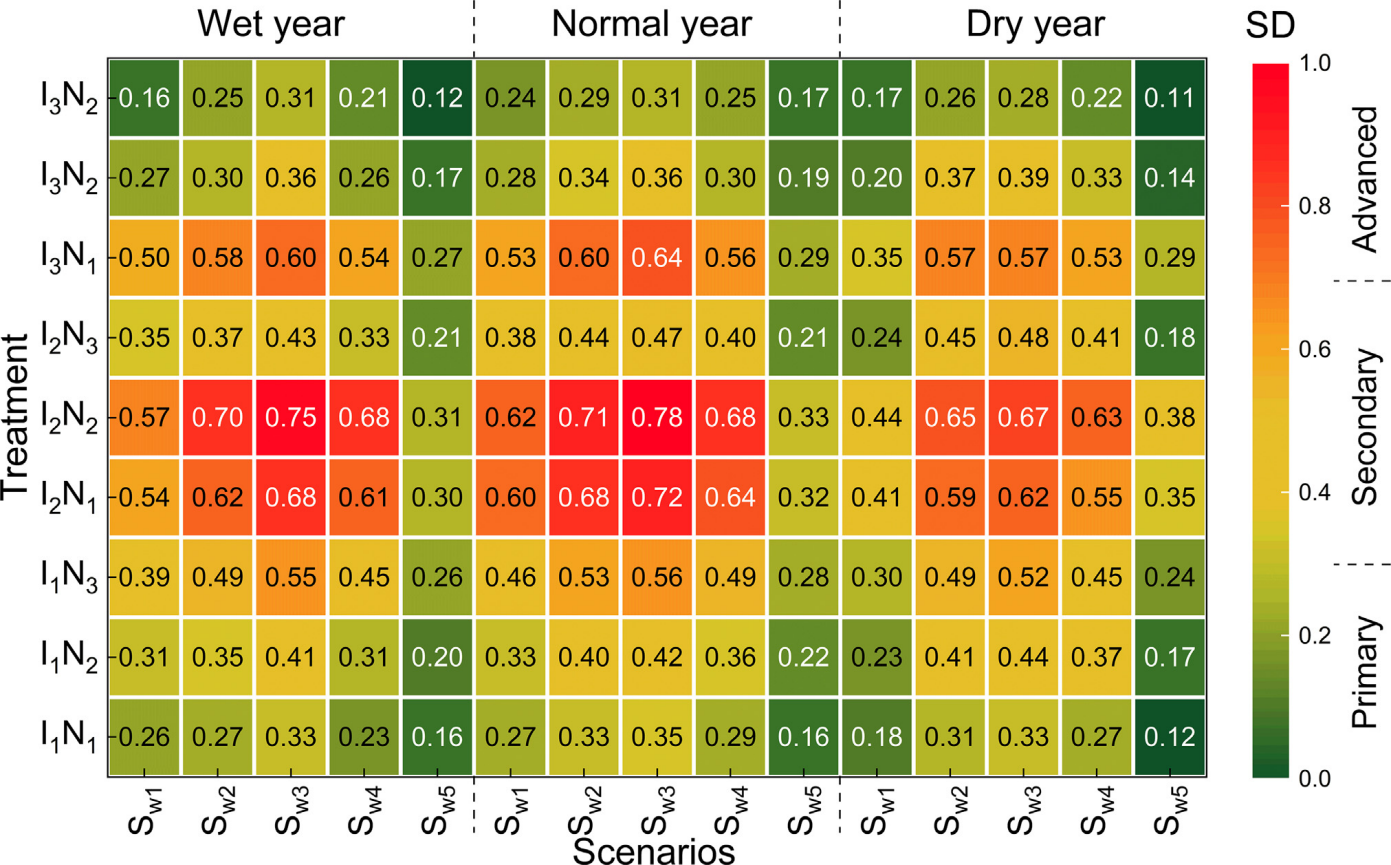
Effect of the straw allocation scheme on the degree of multiobjective coordination

In addition, the degree of coordination was used in this study to analyze the synergistic effect between multiple objectives under different straw allocation schemes^18^ to reduce the influence of conflicting objectives. As shown in Figure 8, the degree of coordination of the treatments under the different straw allocation schemes differed significantly (*p* < 0.05), varying between 0.18 and 0.86. Among them, the I_2_N_2_ treatment was optimal in the flat water years and reached the highest level of coordination, with increases by of 1.16% and 3.27% compared with those in abundant and dry water years, respectively. The other hydrological years were similar. The degree of coordination of each treatment in the S_w2_ and S_w3_ scenarios in different hydrological years exceeded the level of secondary coordination, with increases by of 1.41–7.24% and 2.21–9.62%, respectively, compared with those of the other scenarios. This is because increasing the weight of biochar preparation in cropping ecosystems can improve crop quality while increasing crop yields, increase water use efficiency and reduce GHG emissions, thus contributing to system synergy.

### Multivariate analysis of the effects of farmland water and fertilizer management on farmland ecosystems and drivers

**Figure 9:**
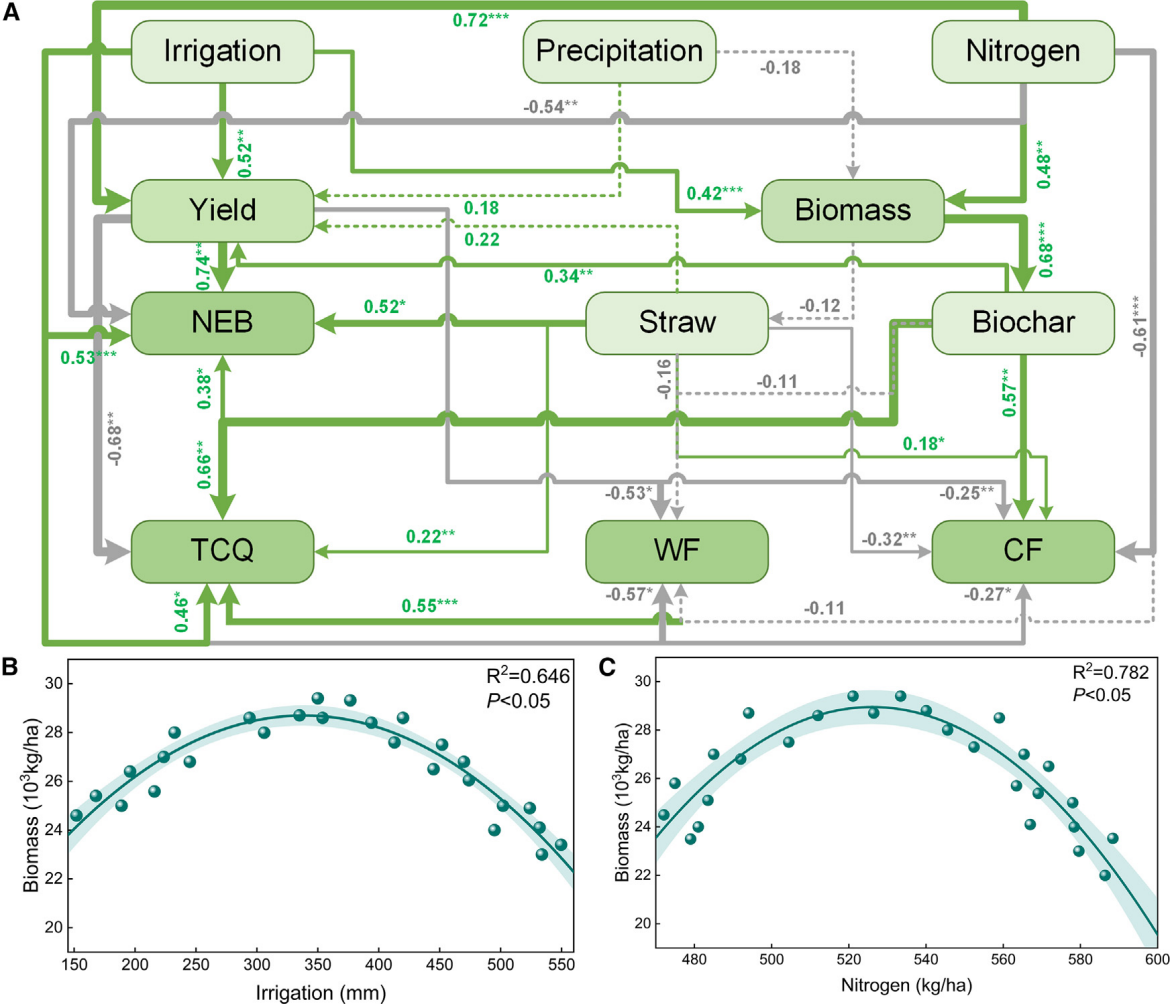
Direct and indirect effects of water and nitrogen application on the sustainable productivity of agroecosystems (A) represents the analysis of water and fertilizer structure equation. (B) represents the impact of irrigation on biomass. (C) represents the effect of nitrogen fertilizer on biomass. Single-headed arrows indicate the direction of causality. Green and gray arrows indicate positive and negative correlations, respectively. The width of each arrow represents the strength of the relationship. The numbers next to the arrows are standardized path coefficients. Shaded areas indicate 95% confidence intervals. Solid lines represent significant effects (∗∗∗*P*<0.001, ∗∗*P*<0.01, ∗*P*<0.05), and dashed lines represent nonsignificant effects (*P*>0.05).

**Figure 10:**
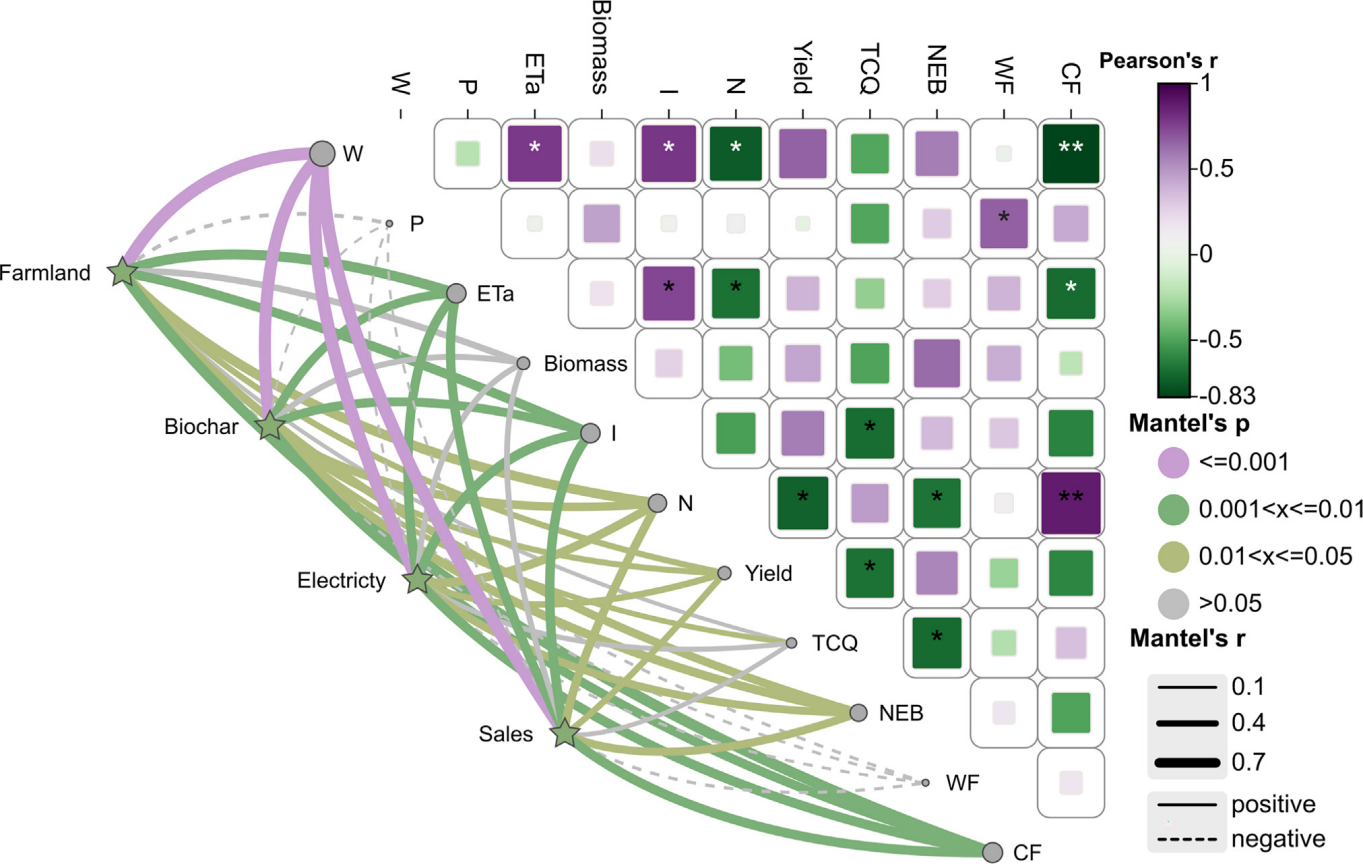
Results of the Mantel test and Pearson correlation analysis between straw allocation schemes and agroecosystems Purple, green, yellow, and gray lines represent significance and nonsignificance at the 0.001, 0.01, and 0.05 levels, respectively (*p* > 0.05). The line width corresponds to the strength of the relationship. Statistics are labeled with “∗∗∗” for *p* < 0.001, “∗∗” for *p* < 0.01, and “∗” for *p* < 0.05. W represents the soil water content; P represents rainfall; ETa represents actual evapotranspiration; I represents irrigation; and N represents nitrogen application. Farmland is the straw return scenario, Biochar is the biochar preparation scenario, Electricity is the straw power generation scenario, and Sales is the straw sale scenario.

Structural equation modeling (SEM) revealed the direct and indirect effects of water and N application on NEB, TCQ, WFs, and CF in the agroecosystem (see Table S2). As shown in Figure 9A, the SEM results revealed that balanced water and N application had significant positive correlations with biomass, yields and TCQ but significant negative correlations (*p* < 0.05) with WF and CF. Straw and biochar application had significant positive correlations (*p* < 0.05) with NEB, TCQ, and CF and negative correlations (*p* < 0.05) with WF. The SEM results also revealed that yield had a significant positive correlation (*p* < 0.05) with NEB and a significant negative correlation (*p* < 0.05) with TCQ, WF, and CF, possibly because an increase in yield led to an increase in the amount of water and nitrogen used, but overapplication led to decreases in TCQ, WFs, and CF. Rainfall had no significant effect (*p* > 0.05) on yield or biomass, which may be due to the greater effects of water and nitrogen application on yield and biomass. In addition, water and nitrogen application had a significant effect (*p* < 0.05) on biomass; both factors indirectly affected straw return and biochar preparation through changes in biomass, and nitrogen fertilizer had a more pronounced direct effect on biomass than did irrigation (Figures 9B and 9C). Mantel’s test revealed (Figure 10) that the straw return, straw power generation and straw sale options in the recycling stage were significantly (*p* < 0.01) influenced by W, ETa, irrigation amount, and CF and were influenced (*p* < 0.05) by nitrogen application, yield, and NEB. Similarly, the biochar preparation regimen during the cycling stage was significantly (*p* < 0.01) influenced by W, ETa, irrigation amount, and CF and was significantly (*p* < 0.05) influenced by nitrogen application, yield, TCQ, and NEB. In addition, P and WF did not significantly (*p* > 0.05) affect the straw return, biochar preparation, straw power generation, or straw sale scenarios in the recycling stage.

## Discussion

Agricultural production is the main source of food and biomass waste, and circular agriculture is regarded as an important strategy to coordinate agricultural production and environmental protection, which can help promote the efficient use of resources and reduce pollution to achieve sustainable development.^19^ In this study, a multidimensional target co-control model was constructed based on field experiments and optimization models to evaluate the impact of water and nitrogen application on farmland ecosystems under different straw utilization schemes and to provide decision support for circular agricultural production and waste management. By introducing randomness and fuzziness, combined with multiobjective stochastic programming and trigonometric intuitionistic fuzzy number methods, the model can more accurately reflect the actual situation and improve the scientific validity and feasibility of decision-making.^20^ From the perspectives of high yield, high quality, water savings, low carbon, and sustainable development, the model comprehensively considers the allocation of water and nitrogen resources and straw resources and balances the effects of water and nitrogen application on yield, resource utilization and GHG emissions under different straw utilization schemes. On the one hand, the model quantifies conflicting goals, such as net economic benefits, the carbon footprint, crop yield, and nutritional quality. On the other hand, the carbon cycle is analyzed in two stages, including carbon emissions from agricultural inputs, carbon sequestration by crops and soils, and carbon fixation from straw return and biochar preparation in the cycle stage. By flexibly adjusting the amount of water and nitrogen and the use of straw, the model can optimize the application of water and nitrogen at different growth stages under different hydrological year systems to increase yield and reduce emissions. Moreover, the goal of the minimizing carbon footprint makes straw return and biochar preparation schemes preferable, promotes carbon sequestration and emission reduction, and further promotes the sustainable development of agricultural ecosystems.

There is a close correlation between water and nitrogen and straw resources in farmland ecosystems, and optimizing this relationship is an effective way to solve the problems of water shortages, excessive application of nitrogen fertilizer and straw reuse.^21^ Most existing studies focus on the effects of straw or biochar combined with water and nitrogen on crop yield, soil fertility and water use efficiency, but evaluations of the combined effects of various straw reuse schemes (such as straw return, biochar preparation, straw power generation, and straw sales) are lacking.^22^ This type of research can help optimize the efficiency of water and nitrogen resources and promote the sustainable development of agriculture. In this study, straw was recycled and reused at multiple levels and converted into biomass for power generation and biochar preparation, thereby promoting crop growth and reducing energy consumption. The farmland ecosystem multiobjective optimization model integrates the circular processes of crop production, biochar preparation, and power generation. It reflects the connection between the water-nitrogen-carbon nexus of different straw utilization schemes and coordinates the relationship between different objectives through multiobjective planning. Compared with traditional agriculture, the reuse of straw in the recycling stage can significantly reduce GHG emissions and promote the sustainable development of agriculture.

On the basis of limited field experimental data, this study verified the practicability and feasibility of the optimal allocation model of water and nitrogen. The optimization results of the water and nitrogen dosages and irrigation water during each growth period of the system under the different hydrological year scenarios are shown in Figure 5. The results revealed that (1) the optimal allocation of water and nitrogen in different hydrological years is an effective measure for coping with extreme climate change, and (2) the reasonable optimal allocation of water resources can effectively prevent the impact of drought on yield during the key growth periods of crops. This result is consistent with the study of Li et al.,^23^ which showed that by increasing the proportion of straw biochar preparation, crop yields can be increased, and GHG emissions and irrigation demand can be reduced. Figure 6 shows that the optimized WF and CF values are reduced by 5.46–2.25% and 13.82–3.37%, respectively, whereas NEB and TCQ are increased by 8.27–21.06% and 4.06–7.63%, respectively. These results differ from those of Yue et al.^24^ because they do not consider factors such as meteorological conditions, differences in soil properties, and carbon emissions between the two phases. In this study, the effects of water and nitrogen application on NEB, TCQ, WF, and CF under different straw utilization schemes were analyzed through life cycle assessment, and the potential of straw reuse in different hydrological years was estimated. The results revealed that the NEB was 13.5–52.6 ×10^3^ yuan/ha, the TCQ was 0.21–0.89, the WF was 3.6–6.4 m^3^/kg, and the CF was 0.54–0.87 kg/kg. This is different from the results of Yue et al.’s^25^ study on optimizing the water-energy-food system, mainly because of the diversity of straw recycling methods and their proportion in the system. Further analysis revealed that increasing the weight of biochar preparation could improve crop yield and quality while reducing GHG emissions and improving system coordination benefits, which was consistent with the results of Rittl et al.^26^ Finally, combined with SEM and Mantel tests, when straw or biochar schemes were preferred, water and nitrogen application was significantly positively correlated with yield and TCQ but negatively correlated with WF and CF. These results are similar to those of Yu et al.,^27^ who reported that straw return and biochar can enhance soil carbon sequestration, increase water retention capacity, and improve the sustainability of the system. However, some of the data in this study are from statistical yearbooks or the literature, and the difference in data in different periods may affect the performance of the model. Future research should consider the impact of factors, such as transportation, crop physiology, and agronomic management on the model.

### Conclusions

In this study, a multiobjective nonlinear planning model for resource allocation in farmland ecosystems with uncertainties was developed to quantitatively analyze the interactions between the integrated water-nitrogen-carbon systems and economic-resource-environmental dimensions. The model integrates the optimal allocation of water, nitrogen fertilizer, and straw resources in the initial and recycling stages of the system and balances the competing relationships among economic, resource, and environmental objectives. The modeling framework includes methods for constructing the objective function of the integrated quality of system crops, determining the weights on the basis of the game-theoretic combinatorial assignment method, and assessing the sustainability of the system through the degree of coordination. The main innovations and contributions of this study are as follows: (1) an integrated optimization model framework based on integrated water-nitrogen-carbon systems is provided for the sustainable management of agricultural water, nitrogen fertilizer, and straw resources; (2) the model quantifies the relationship between water and nitrogen resources and the comprehensive system quality, especially considering the conflicting relationship between balanced crop yields and quality; and (3) the consideration of abundant yields and high-quality, water-saving, low-carbon, and sustainable perspectives provides a rational decision scheme for water, nitrogen, and straw resource allocation that balances economic, resource, and environmental objectives.

The model was applied to data from a farmland experiment to study agricultural resource allocation and the water-nitrogen-carbon association in farmland ecosystems, and the main conclusions were as follows: (1) rational water and nitrogen resource optimization in different hydrological years can effectively avoid the impacts of rainfall on crop production during the critical reproductive period of the crop. (2) When the water and nitrogen use in the tomato-corn-soybean agroecosystem was 196-157-123 mm and 207-236-84 kg/ha, respectively, the optimal values of the water and carbon footprints of the treatments were reduced by 2.25–5.46% and 3.37–13.82%, respectively, compared with the status quo values of local farmers, and the economic benefits and overall quality were increased by 8.27–21.06% and 4.06–7.63%, respectively. (3) When the weights of straw return (Sw2) and biochar preparation from straw (Sw3) in the recycling stage increased, the coordination level of each treatment in different hydrological years increased by 1.41–7.24% and 2.21–9.62%, respectively, compared with those in the other scenarios, resulting in an advanced level of coordination. (4) The application of water and nitrogen under the different straw utilization scenarios had significant direct or indirect effects. The modeling framework proposed in this study can be applied to similar cropping system sustainability studies. However, application of the framework in circular farming systems requires the incorporation of more integrated water-nitrogen-carbon systems as well as expansion of the matter and energy transport pathways in the framework to improve its practicality and reliability.

### Limitations of the study

While the findings of this study provide valuable insights into the optimization of straw, water, and nitrogen resources in agricultural ecosystems, several limitations should be acknowledged. First, the study was conducted within a specific agroecosystem (tomato-corn-soybean) and may not be directly applicable to other crop systems or regions with differing environmental and soil conditions. The model used to simulate the interactions between water, nitrogen, and carbon may not fully capture all complex, real-world dynamics, such as unforeseen environmental variables or long-term soil health impacts, which could influence the outcomes. Additionally, the study assumes uniformity in farmer practices, whereas real-world application may face variability in implementation. The economic benefits also depend on local market conditions, which can fluctuate. Future studies could benefit from expanding the scope to include more diverse ecosystems and further validation of model predictions across different agroecosystems. Additionally, methods such as remote sensing or field-based trials could complement this modeling approach to enhance real-world applicability.

## Resource availability

### Lead contact

Further information and requests for resources and reagents should be directed to and will be fulfilled by the lead contact, Mo Li (limo0828@neau.edu.cn).

### Materials availability

This study did not generate new materials.

### Data and code availability

• All data reported in this paper will be shared by the lead contact upon request.
• This paper does not report original code.
• Any additional information required to reanalyze the data reported in this paper is available from the lead contact upon request.

## Acknowledgments

Supports from the 10.13039/501100001809National Natural Science Foundation of China (grant no.52222902, and 52479035) are acknowledged.

## Author contributions

Conceptualization, M.L. and P.Z.; methodology, M.L., P.Z., and X.W.; Investigation, M.L., P.Z., and A.Y.; writing—original draft, P.Z. and Y.S.; writing—review and editing, M.L. and Y.S.; funding acquisition, M.L.; resources, Q.F. and M.L.; supervision, P.Z., Y.S., and A.Y.

## Declaration of interests

The authors declare that they have no competing interests.

## Declaration of generative AI and AI-assisted technologies

During the preparation of this work, the author(s) used ChatGPT 3.5 in order to improve the grammar and readability. After using this tool, the author(s) reviewed and edited the content as needed and take(s) full responsibility for the content of the publication.

## STAR★Methods

### Key resources table

**Table:** 

REAGENT or RESOURCE	SOURCE	IDENTIFIER
**Software and algorithms**		

Origin	Origin 2023	https://www.originlab.com/2023
Python	Python 3.9.0	https://www.python.org/downloads/release/python-390/

### Experimental model and study participant details

There are no experimental model or study participants to include in this paper.

### Method details

#### Study site

The experiments were carried out from April–October 2021 and 2022 in the Modern Agricultural High-Tech Demonstration Park in Harbin city, Heilongjiang Province. The test site is located at 45°63′N latitude and 125°44′E longitude, with an average elevation of 162 m. It has a cold-temperate continental climate with warm and rainy summers and cold and dry winters, with an average multiyear temperature of 3.46°C and a frost-free period of approximately 128 d. The average annual rainfall is 553.2 mm, which is concentrated mainly from June to August, and the average annual evaporation is 796 mm. The greenhouse was 50 m long, 8.5 m wide and 4 m high. The soil type was black, and the basic physicochemical properties of the soil were as follows: pH value: 7.08; bulk density, organic matter, total nitrogen, total phosphorus, and total potassium: 1.20, 41.26, 1.85, 1.16, and 19.99 g/kg; and alkaline dissolved nitrogen, quick-acting phosphorus, and quick-acting potassium: 159.21, 28.57, and 98.62 mg/kg, respectively. The field water holding capacity of the 0–60 cm soil layer was 30.54%, and the wilting water content was 12.72% (mass ratio). The study area is shown in Figure 1.

#### Experimental design

To determine the economic and environmental effects of water and nitrogen rationing with straw and biochar application on agroecosystems, tomato, corn and soybean were selected for experiments based on adequate irrigation (*I*) and locally recommended nitrogen (*N*) application rates (*CF*_*T*_: 200 kg/ha, *CF*_*C*_: 240 kg/ha and *CF*_*S*_: 120 kg/ha). Irrigation, nitrogen and additives (biochar and straw) were selected as the experimental factors, with three irrigation levels (*I*_*1*_: *I*, *I*_*2*_: 0.75*I* and *I*_*3*_: 0.5*I*), three nitrogen levels (*N*_*1,T-C-S*_: 1.5*CF*_*T-C-S*_, *N*_*2,T-C-S*_: *CF*_*T-C-S*_ and *N*_*3,T-C-S*_: 0.5*CF*_*T-C-S*_; *CF* is the local recommended nitrogen application rate) and three additive levels (*B*_*1*_*+S*_*1,T-C-S*_: 30(0)-60(0)-40(0) t/ha, *B*_*2*_*+S*_*2,T-C-S*_: 15(15)-30(30)-20(20) t/ha and *B*_*3*_*+S*_*3,T-C-S*_: 0(30)-0(60)-0 (40) t/ha). A total of nine treatments, each replicated three times, were arranged in randomized blocks (Table 1). In the experiment, phosphorus fertilizer (P_2_O_5_), potash fertilizer (K_2_O), biochar and straw were applied in a one-time basal application, and nitrogen fertilizer was applied at a basal-topdressing ratio of 1:2. To prevent side-row effects in the plot, protective rows were set up around the perimeter, while water traps buried at a depth of 1 m were used to prevent side seepage of water and fertilizer. Drip irrigation was used to apply both water and fertilizer to each crop, with an irrigation frequency of 7/d. The other field management measures used were the same as those used by local farmers. The biochar used in this study was obtained by pyrolyzing corn stover for 5–8 h in a continuous vertical biochar furnace at 400–600°C; the material had a pH value of 10.24, a specific surface area of 9 m^2^/g, a bulk weight of 0.19 g/cm^3^, a total porosity of 67.03%, a water-holding porosity of 61.17%, a fixed carbon content of 650 g/kg, a quick-acting phosphorus content of 10.21 g/kg, a quick-acting potassium content of 55.65 g/kg, a nitrate content of 650 g/kg, a nitrate nitrogen content of 2.26 mg/kg, and an ammonium nitrogen content of 1.11 mg/kg.

#### Observations and measurements

Fifteen plants were randomly selected from each corn and soybean plot, dried and threshed to measure the number of kernels per ear, kernel weight per ear, and thousand kernel weight, which were finally converted to the kernel yield per unit area. The greenhouse tomatoes were weighed at harvest to calculate the yield of each test plot. Three representative plants from each test plot were selected during the crop harvesting period, and the samples were dried to a constant weight after surface dirt was removed. The dried samples were weighed separately, and the moisture contents of the seeds were determined. Meteorological monitoring stations were set up in the large field and greenhouse to automatically record parameters such as atmospheric pressure, temperature, photosynthetically active radiation, relative humidity, and solar radiation.

For yield comparisons between different crop products, the equivalent yields were calculated by multiplying the crop yields by the market price of tomatoes in the same year.^28^ The equivalent yields for each cropping system were as follows:

(Equation 1) $$EY=Yield\cdot P/P_{tomato}$$

where Yield is the equivalent yield (kg/ha); $P_{c}$ is the price of the selected crop c (yuan/kg); and $P_{tomato}$ is the price of tomato (yuan/kg).

Soil samples were collected from the 0–40 cm soil layer at each fertility stage at the beginning of the experiment. The soil bulk weight was determined via the ring knife method; the soil organic carbon content was determined via oxidation with concentrated sulfuric acid potassium dichromate; and the soil moisture content was determined using a Diviner 2000 soil moisture profiler and a revised drying method. A field water holding capacity of 90% was used as the upper irrigation limit for the fully irrigated treatment as follows:

(Equation 2) $$I=10\cdot \left(0.9\theta_{Fc}-\theta_{V}\right)\cdot Z_{r}\cdot 0.6$$

where 10 is a unit conversion factor; *I* is the amount of irrigation water (mm) for the fully irrigated treatment; $\theta_{Fc}$ is the field water holding capacity (cm^3^/cm^3^); $\theta_{V}$ is the soil water content before irrigation (cm^3^/cm^3^); $Z_{r}$ is the designed depth of the wetted layer (cm); and 0.6 is the wetting ratio.

#### Methodology

##### Model development

Farmland water and nitrogen management directly affects NEB, TCQ, WF and CF, and complex mutual effects exist among these indicators. Scientifically supported fertilization and irrigation measures can not only increase crop yields but also effectively reduce carbon emissions, enhance the efficiency of water and nitrogen resource utilization, and improve the quality of agricultural products. Optimizing water and nitrogen management can effectively balance these interrelated goals. In addition, water and nitrogen application increases crop yields while producing large amounts of straw. On the basis of the principles of reduction, recycling, reuse, and standardization in circular agriculture, the straw collected in the initial and recycling stages is utilized for crushing and returning to the field, biochar preparation, power generation, and sales. Specifically, part of the straw collected in the initial stage is burned and gasified to generate electricity, which is then consumed in the recycling stage of agricultural production to power shuttering machines, lighting, and irrigation. Another portion of the straw collected in the initial stage is gasified and pyrolyzed to prepare biochar, which is then applied to the fields in the recycling stage to increase yield, improve crop quality, and increase the carbon sequestration and emission reduction capacity of the soil. Additionally, part of the straw collected in the initial stage is crushed and returned to the field, which helps reduce the cost of agricultural production. Another portion of the straw collected in the initial stage and the straw collected in the recycling stage are sold directly, thus increasing economic benefits. When sufficient electricity and biochar can be produced in the initial stage to meet agricultural demands in the recycling stage, the remaining straw can be sold to increase economic benefits; in contrast, when insufficient straw is collected in the initial stage to meet demand under the straw diversification scheme, additional straw can be purchased from outside to meet recycling needs. The straw recycling scheme is shown in Figure 2.

Therefore, this study established a model framework for the green and efficient regulation of agricultural resources based on a combination of experimental research and optimization modeling (Figure 2), with the integrated water‒nitrogen‒carbon systems of the agroecosystem as the core. The aim is to increase food production, improve crop quality and ensure economic income for farmers while reducing resource input costs and minimizing environmental pollution. The framework integrates three main components. First, triangular intuitionistic fuzzy numbers (TIFNs) are used to address the vagueness and uncertainty of system parameters and increase the robustness of the regulatory modeling framework. Moreover, Monte Carlo stochastic simulation is used to analyze the effect of precipitation on the stability of the model framework in different hydrological years. Second, the integrated quality of tomato‒corn‒soybean agroecosystems under the straw–biochar return program is determined in response to water and nitrogen application during the whole reproductive period. Finally, through comprehensive consideration of the cyclic relationship between straw utilization and water and nitrogen resource allocation in agroecosystems, the green and efficient regulation of agricultural resources in agroecosystems can be realized from the perspectives of high yield, high quality, water conservation, low carbon and sustainability. By balancing the synergistic development of multiple system objectives (NEB, TCQ, WF and CF), the sustainable development of straw resource recycling, water and nitrogen resource optimization, food security and ecological environmental protection can be achieved. The findings provide technical support and a scientific reference for sustainable agricultural development and environmental protection.

##### Optimization module

The model contains four objective functions—maximum economic benefit, maximum comprehensive quality, minimum water footprint and minimum carbon footprint—with constraints on electricity, water, nitrogen fertilizer, biochar, straw, and straw allocation weight and nonnegative constraints. The decision variables include the straw utilization method, the optimal allocation proportion of straw, and the optimal allocation scheme of water and nitrogen resources.

##### Net economic benefit function

The NEB of an agroecosystem is the economic benefit obtained on the basis of the economic effects achieved, which can be calculated as the difference between the economic income from agriculture and the cost of cultivation, as shown below:

(Equation 3) $$\max\;F_{NEB,d}=F_{REV,d}-F_{\mathrm{COS},d}$$

where $F_{NEB,d}$ is the objective function of the NEB of treatment d (10^3^ yuan/ha); $F_{REV,d}$ is the agricultural economic income of treatment d (10^3^ yuan/ha); and $F_{\mathrm{COS},d}$ is the cultivation cost of treatment d (10^3^ yuan/ha).

Agricultural economic income includes income from food for the entire system, unregenerated straw from the initial stage, all straw from the recycling stage, and the sale of electricity generated in the initial stage, as shown below:

(Equation 4) $$F_{REV,d}=\sum_{c=1}^{C}P_{crop,dc}A_{area,dc}^{st}Y_{dc}^{st}+\sum_{c=1}^{C}P_{crop,dc}A_{area,dc}^{st+1}Y_{dc}^{st+1}+S_{straw,d}^{st}+S_{straw,d}^{st+1}+E_{ele,d}^{st+1}$$

where st is the initial stage; st+1 is the recycling stage; $P_{crop,dc}$ is the price of crop c under treatment d (10^3^ yuan/kg); $A_{area,dc}^{st}$ and $A_{area,dc}^{st+1}$ are the planted areas of crop c under treatment d in stages st and st+1, respectively (ha); $Y_{dc}^{st}$ and $Y_{dc}^{st+1}$ are the yields of crop c under treatment d in stages st and st+1, respectively (kg/ha); $S_{straw,d}^{st}$ and $S_{straw,d}^{st+1}$ are the economic benefits of straw in stages st and st+1, respectively (10^3^ yuan/ha); and $E_{ele,d}^{st+1}$ is the economic benefit of electricity generation in stage st (10^3^ yuan/ha).

The Jensen model, which is a commonly used model of water production based on the fertility stage, also accounts for the effect of the interaction of water and nitrogen on yield at each fertility stage,^29^ as shown below:

(Equation 5) $$Y_{dc}^{st}=Y_{dc}^{m,st}\prod_{t=1}^{T}{\left(ET{\left(t\right)}_{a,dc}^{st}/ET{\left(t\right)}_{m,dc}^{st}\right)}^{\lambda {\left(t\right)}_{c}}$$

(Equation 6) $$Y_{dc}^{m,st}=a_{crop,c}^{st}{\left(N_{crop,dc}^{st}\right)}^{2}+b_{crop,c}^{st}N_{crop,dc}^{st}+c_{crop,c}^{st}$$

where t is the number corresponding to the crop fertility stage; T is the total number of crop fertility stages; $Y_{dc}^{m,st}$ is the actual yield (kg/ha) of crop c under treatment d in stage st; $Et{\left(t\right)}_{a,dc}^{st}$ is the actual evapotranspiration (mm) of crop c under treatment d in stage st; $Et{\left(t\right)}_{m,dc}^{st}$ is the potential evapotranspiration (mm) of crop c under treatment d in stage st; $\lambda {\left(t\right)}_{c}$ is the moisture sensitivity index of crop c in stage st; $N_{crop,dc}^{st}$ is the amount of nitrogen applied to crop c under treatment d in stage st (kg/ha); and $a_{crop,c}^{st}$, $b_{crop,c}^{st}$ and $c_{crop,c}^{st}$ are coefficients in the production function for crop c.

The actual crop evapotranspiration is related to various quantitative water parameters,^23^ and the field water balance is expressed as follows:

(Equation 7) $$\Delta W{\left(t\right)}_{dc}^{st}=P{\left(t\right)}_{dc}^{st}+I{\left(t\right)}_{dc}^{st}+G{\left(t\right)}_{dc}^{st}-D{\left(t\right)}_{dc}^{st}-R{\left(t\right)}_{dc}-Et{\left(t\right)}_{a,dc}^{st}$$

where $P{\left(t\right)}_{dc}^{st}$ is the effective rainfall (mm) for crop c in stage t; $I{\left(t\right)}_{dc}^{st}$ is the amount of irrigation (mm) for crop c in fertility stage t; $G{\left(t\right)}_{dc}^{st}$ is the amount of groundwater recharge (mm) for crop c in fertility stage t; $D{\left(t\right)}_{dc}^{st}$ is the amount of deep seepage (mm) for crop c in fertility stage t; $R{\left(t\right)}_{dc}^{st}$ is the amount of surface runoff (mm) for crop c in fertility stage t; and $\Delta W{\left(t\right)}_{dc}^{st}$ is the amount of change in soil moisture (mm).

Crop yields during the recycling stage are affected by the straw and biochar added to the farmland, in addition to water and nitrogen, as shown below:

(Equation 8) $$Y_{dc}^{st+1}=\left(1+\alpha_{yield,c}\right)Y_{dc}^{st}$$

(Equation 9) $$\alpha_{yield,c}=a_{yield,c}{\left(y_{biochar,dc}^{st+1}\right)}^{2}+b_{yield,c}{\left(y_{straw,dc}^{st+1}\right)}^{2}+c_{yield,c}y_{biochar,dc}^{st+1}y_{straw,dc}^{st+1}+d_{yield,c}y_{biochar,dc}^{st+1}+e_{yield,c}y_{straw,dc}^{st+1}+f_{yield,c}$$

(Equation 10) $$y_{straw,dc}^{st+1}=pr_{straw}Q_{biomass,dc}^{st}$$

where $\alpha_{yield,c}$ is the yield coefficient of crop c; $y_{biochar,dc}^{st+1}$ and $y_{straw,dc}^{st+1}$ are the actual amounts of biochar and straw (kg) used in stage st+1 for crop c under treatment d, respectively; $a_{yield,c}$, $b_{yield,c}$, $c_{yield,c}$, $d_{yield,c}$, $e_{yield,c}$, and $f_{yield,c}$ are the yield coefficients of crop c; $pr_{straw}$ is a weighting factor for the amount of straw returned to the field; and $Q_{biomass,dc}^{st}$ is the amount of biomass in stage st for crop c under treatment d (kg/ha).

The biomass produced in the initial stage is generated from agricultural residues, as shown below:

(Equation 11) $$Q_{biomass,dc}^{st}=A_{area,dc}^{st}Y_{area,dc}^{st}\omega_{c}\xi_{c}\psi_{energy,c}$$

where $\omega_{c}$ is the grass-to-grain ratio for crop c; $\xi_{c}$ is the straw recovery coefficient for crop c; and $\psi_{energy,c}$ is the ratio of straw to biomass.

The biochar returned to the field in the cycle stage mainly consists of two parts: one is the byproduct of the power generation process, and the other is the biochar specially prepared for agricultural applications. Specifically, the biochar produced in the power generation process is derived from biomass collected in the initial stage and converted through high-temperature pyrolysis and gasification processes. On the other hand, biochar specially prepared for agricultural use is obtained by gasification and pyrolysis of organic matter such as straw collected in the initial stage, as follows^23^:

(Equation 12) $$y_{biochar,dc}^{st+1}=\left(\eta_{gas}pr_{biochar}Q_{biomass,dc}^{st}-\eta_{gas-ele}Q_{ele-gas,dc}^{st}\right)+\eta_{pyr}pr_{biochar}Q_{biomass,dc}^{st}$$

where $y_{biochar,dc}^{st+1}$ is the amount of biochar (kg/ha) for crop c under treatment d in stage st+1; $Q_{ele-gas,dc}^{st}$ is the electricity generated from straw gasification for crop c under treatment d in stage st (kW.h); $\eta_{gas-ele}$ is the conversion factor for electricity; $\eta_{gas}$ is the coefficient of straw gasification; $\eta_{pyr}$ is the coefficient of thermal cracking of straw; and $pr_{biochar}$ is the weight of straw used for the preparation of biochar.

In the initial stage, the system's electrical energy revenues are generated primarily from straw, as shown below:

(Equation 13) $$E_{ele,d}^{st}=\sum_{c=1}^{C}P_{ele-sal}\left(Q_{ele-com,dc}^{st}+Q_{ele-gas,dc}^{st}\right)$$

where $P_{ele-sal}$ is the selling price of electricity (10^3^ yuan/kW.h).

The source of electricity in the system in the cycle stage is mainly biomass collected and processed in different ways in the initial stage. Specifically, the biomass collected in the initial phase is converted into electricity after treatment technologies such as gasification and direct combustion. Among these treatment technologies, gasification technology and direct combustion technology are the two most common and promising technologies at present, and they have significant advantages in terms of improving energy efficiency and reducing emissions, as follows^25^:

(Equation 14) $$Q_{ele-com,dc}^{st}=H\nu_{c}\zeta_{the}\rho_{com}pr_{ele}Q_{biomass,dc}^{st}/3.6$$

(Equation 15) $$Q_{ele-gas,dc}^{st}=H\nu_{c}\zeta_{the}\zeta_{com}\rho_{gas}pr_{ele}Q_{biomass,dc}^{st}/3.6$$

where $Q_{ele-com,dc}^{st}$ is the electricity (kW.h) generated by the combustion of crop c under treatment d in stage st; $H\nu_{c}$ is the heating value (kJ/kg); $pr_{ele}$ is the proportion of straw used for power generation; $\zeta_{the}$ is the thermal efficiency of the internal combustion engine; $\zeta_{com}$ is the coefficient of conversion of mechanical energy into electrical energy; $\rho_{com}$ and $\rho_{gas}$ are the combustion and gasification ratios, respectively; and 3.6 is the energy conversion factor.

The system's revenue from straw sales includes revenues from the sale of unregenerated straw in the initial stage and all straw in the recycling phase, as shown below:

(Equation 16) $$S_{straw,d}^{st}=\sum_{c=1}^{C}P_{straw,dc}A_{area,dc}^{st}Y_{dc}^{st}\omega_{c}\zeta_{c}pr_{sell}Q_{biomass,dc}^{st}$$

(Equation 17) $$S_{straw,d}^{st+1}=\sum_{c=1}^{C}P_{straw,dc}A_{area,dc}^{st+1}Y_{dc}^{st+1}\omega_{c}\zeta_{c}$$

where $P_{straw,dc}$ is the selling price (10^3^ yuan/kg) of straw from crop c under treatment d and $pr_{sell}$ is the proportion of straw sold.

The cost of agricultural cultivation in agroecosystems mainly includes the costs of materials, electricity and irrigation in the production process, as shown below:

(Equation 18) $$F_{\mathrm{COS},d}=\sum_{c=1}^{C}A_{area,dc}^{st}\left(C_{mat.dc}^{st}+C_{ele.dc}^{st}+C_{wat.dc}^{st}\right)+\sum_{c=1}^{C}A_{area,dc}^{st+1}\left(C_{mat.dc}^{st+1}+C_{ele.dc}^{st+1}+C_{wat.dc}^{st+1}\right)$$

where $C_{mat,dc}^{st}$ and $C_{mat,dc}^{st+1}$ denote the material cost (10^3^ yuan/kg) of crop c under treatment d in stages st and st+1, respectively; $C_{ele,dc}^{st}$ and $C_{ele,dc}^{st+1}$ denote the electricity cost (10^3^ yuan/kW.h) of crop c under treatment d in stages st and st+1, respectively; and $C_{wat,dc}^{st}$ and $C_{wat,dc}^{st+1}$ denote the irrigation cost of crop c under treatment d in stages st and st+1, respectively (10^3^ yuan/m^3^).

The material costs in the agricultural production process mainly include fertilizer, pesticide, seed, agricultural film, diesel fuel, purchased straw, purchased biochar, and labor costs, as shown below:

(Equation 19) $$C_{mat,dc}^{st}=(P_{see}D_{see,dc}+P_{fer}D_{fer,dc}+P_{pes}D_{pes,dc}+P_{fue}D_{fue,dc}+P_{fil}D_{fil,dc}+P_{lab}D_{lab,dc})A_{area,dc}^{st}$$

(Equation 20) $$C_{mat,dc}^{st+1}=C_{mat,dc}^{st}+y_{biochar,dc}^{st+1}P_{biochar}A_{area.dc}^{st+1}+y_{straw,dc}^{st+1}P_{straw}A_{area.dc}^{st+1}$$

where $P_{see}$, $P_{fer}$, $P_{pes}$, $P_{fue}$, $P_{fil}$, $P_{lab}$, $P_{biochar}$, and $P_{straw}$ denote the seed (10^3^ yuan/kg), fertilizer (10^3^ yuan/kg), pesticide (10^3^ yuan/kg), diesel (10^3^ yuan/kg), agrofilm (10^3^ yuan/kg), labor (10^3^ yuan/h), and biochar purchasing (10^3^ yuan/kg) prices, respectively; $D_{see,dc}$, $D_{fer,dc}$, $D_{pes,dc}$, $D_{fue,dc}$, $D_{fil,dc}$, and $D_{lab,dc}$ denote the seed (kg/ha), fertilizer (kg/ha), pesticide (kg/ha), diesel fuel (kg/ha), agricultural film (kg/ha), and labor force (h/ha) usage amounts, respectively.

The power cost of the system includes the power consumption of the rollers, lighting and irrigation, as shown below:

(Equation 21) $$C_{ele,dc}^{st}=P_{ele-pur,d}A_{area,dc}^{st}\left(D_{ele-lig,dc}^{st}H_{wat-lig,dc}^{st}+D_{ele-irr,dc}^{st}\sum_{t=1}^{T}I_{dc}^{st}+D_{ele-rol}^{st}\tau_{rol}\right)$$

(Equation 22) $$C_{ele,dc}^{st+1}=P_{ele-pur,d}A_{area,dc}^{st+1}\left(D_{ele-lig,dc}^{st+1}H_{wat-lig,dc}^{st+1}+D_{ele-irr,dc}^{st+1}\sum_{t=1}^{T}I_{dc}^{st+1}+D_{ele-rol}^{st+1}\tau_{rol}\right)$$

where $P_{ele-pur,d}$ is the purchase price of electricity (10^3^ yuan/kW.h); $D_{ele-lig,dc}^{st}$ and $D_{ele-irr.dc}^{st}$ denote the electricity consumption for lighting (kW/h) and irrigation (kW.h/m^3^) of crop c under treatment d in stage st, respectively; $H_{ele-lig,dc}^{st}$ denotes the duration of illumination (h/ha) under treatment d in stage st; $D_{ele-lig,dc}^{st+1}$ and $D_{ele-irr,dc}^{st+1}$ denote the electricity consumption (kW.h/m^3^) for lighting and irrigation under treatment d in stage st+1, respectively; $H_{wat-sur,dc}^{st+1}$ denotes the duration of illumination (h/ha) under treatment d in stage st+1; $D_{ele-rol,dc}^{st}$ and $D_{ele-rol,dc}^{st+1}$ denote the electricity consumption (kW/ha) under treatment d in stages st and st+1, respectively; and $\tau_{rol}$ is the efficiency the greenhouse rollers.

The cost of water in the system includes the expenditures for irrigation in the two stages, as shown below:

(Equation 23) $$C_{wat,dc}^{st}=P_{wat}\sum_{t=1}^{T}I_{dc}^{st}$$

(Equation 24) $$C_{wat,dc}^{st+1}=P_{wat}\sum_{t=1}^{T}I_{dc}^{st+1}$$

where $P_{wat}$ is the purchase price of irrigation water (10^3^ yuan/m^3^).

##### System’s comprehensive quality function

The crop quality of a system also needs to be taken into account when considering the economic benefits of agroecosystems, as crop quality is directly related to agricultural output and market competitiveness. High-quality crops also help reduce after-sale costs and minimize waste and return rates, which in turn indirectly contributes to the economic benefits of the system.^28^ Therefore, in this study, on the basis of the measured values of crop quality indices (Figure 3), the objective function relating water and nitrogen application and the crop quality of the system under different straw utilization scenarios was constructed via multiple regression analysis as follows:

(Equation 25) $$\max\;F_{TCQ,d}=0.5\sum_{c=1}^{C}\omega_{crop,dc}^{st}Cq_{crop.dc}^{st}+0.5\sum_{c=1}^{C}\omega_{crop,dc}^{st+1}Cq_{crop.dc}^{st+1}$$

(Equation 26) $$Cq_{crop,dc}^{st}=ac_{crop,c}^{st}{\left(N_{crop.dc}^{st}\right)}^{2}+bc_{crop,c}^{st}{\left(\sum_{t=1}^{T}I_{crop.dc}^{st}\right)}^{2}+cc_{crop,c}^{st}N_{crop.dc}^{st}+dc_{crop,c}^{st}\sum_{t=1}^{T}I_{crop.dc}^{st}+ec_{crop,c}^{st}N_{crop.dc}^{st}\sum_{t=1}^{T}I_{crop.dc}^{st}+fc_{crop.c}^{st}$$

where $Cq_{crop,dc}^{st}$ is the composite crop quality of crop c under treatment d in stage st; $\omega_{crop,dc}^{st}$ and $\omega_{crop,dc}^{st+1}$ are the weights of crop c under treatment d in the system in stages st and st+1; and $ac_{crop,c}^{st}$, $bc_{crop,c}^{st}$, $cc_{crop,c}^{st}$, $dc_{crop,c}^{st}$, $ec_{crop,c}^{st}$, and $fc_{crop,c}^{st}$ are coefficients in the quality function of crop c in stage st.

(Equation 27) $$Cq_{crop,dc}^{st+1}=\left(1+\alpha_{TCQ,c}\right)Cq_{crop,dc}^{st}$$

(Equation 28) $$\alpha_{TCQ,c}=a_{TCQ,c}{\left(y_{biochar,dc}^{st+1}\right)}^{2}+b_{TCQ,c}{\left(y_{straw,dc}^{st+1}\right)}^{2}+c_{TCQ,c}y_{biochar,dc}^{st+1}y_{straw,dc}^{st+1}+d_{TCQ,c}y_{biochar,dc}^{st+1}+e_{TCQ,c}y_{straw,dc}^{st+1}+f_{TCQ,c}$$

where $\alpha_{TCQ,c}$ is the combined nutrient quality improvement coefficient for crop c and $a_{TCQ,c}$, $b_{TCQ,c}$, $c_{TCQ,c}$, $d_{TCQ,c}$, $e_{TCQ,c}$ and $f_{TCQ,c}$ denote the quality coefficients for crop c.

##### System’s carbon footprint function

CF is used to assess the GHG emissions of agroecosystems to reduce the environmental impacts of agricultural production while ensuring sustainable agricultural development. The CF in cyclic systems is categorized into carbon emissions from agricultural products and carbon emissions and sequestration in the crop‒soil system,^30^ as shown below:

(Equation 29) $$\min\;F_{CF,d}=\sum_{c=1}^{C}\left(f_{TCE,dc}-f_{CS,dc}\right)/\left(Y_{dc}^{st}+Y_{dc}^{st+1}\right)$$

where $F_{CF,d}$ is the carbon footprint for the production of crop c under treatment d (kg/ha); $F_{TCE,dc}$ is the total GHG emissions of crop c under treatment d (kg/ha); and $f_{CS,dc}$ is the carbon sequestered by crop c under treatment d (kg/ha).

The total GHG emissions in the agroecosystem include CO_2_, CH_4_, and N_2_O in both production stages, as shown below:

(Equation 30) $$\begin{array}{l} f_{TCE,dc}=\left(En_{CO_{2},dc}^{st}+28En_{CH_{4},dc}^{st}+298En_{N_{2}O,dc}^{st}\right)+\\ \left(En_{CO_{2},dc}^{st+1}+28En_{CH_{4},dc}^{st+1}+298En_{N_{2}O,dc}^{st+1}\right) \end{array}$$

where $En_{CO_{2},dc}^{st}$, $En_{CH_{4},dc}^{st}$ and $En_{N_{2}O,dc}^{st}$ denote the CO_2_ (kg/ha), CH_4_ (kg/ha) and N_2_O emissions (kg/ha) of crop c under treatment d in stage st, respectively; $En_{CO_{2},dc}^{st+1}$, $En_{CH_{4},dc}^{st+1}$, and $En_{N_{2}O,dc}^{st+1}$ denote the CO_2_ (kg/ha), CH_4_ (kg/ha) and N_2_O emissions (kg/ha) of crop c under treatment d in stage st+1, respectively; and 28 and 298 denote the conversion factors for CH_4_ and N_2_O, respectively.

CO_2_ emissions are attributed to inputs such as fertilizers, pesticides, seeds, agricultural films, diesel fuel, and labor costs in both stages, and the application of biochar and straw in the recycling stage helps mitigate GHG emissions, as shown below:

(Equation 31) $$\begin{array}{l} En_{CO_{2},dc}^{st}=A_{area,dc}^{st}(\sigma_{see}D_{see,dc}+\sigma_{fer}D_{fer,dc}+\sigma_{pes}D_{pes,dc}+\sigma_{fue}D_{fue,dc}+\sigma_{fil}D_{fil,dc}+\sigma_{lab}D_{lab,dc}) \end{array}$$

(Equation 32) $$En_{CO_{2},dc}^{st+1}=\left(1+\vartheta_{CO_{2},dc}\right)En_{CO_{2},dc}^{st}$$

(Equation 33) $$\vartheta_{CO_{2},dc}=a_{CO_{2},dc}{\left(y_{biochar,dc}^{st+1}\right)}^{2}+b_{CO_{2},dc}{\left(y_{straw,dc}^{st+1}\right)}^{2}+c_{CO_{2},dc}y_{biochar,dc}^{st+1}y_{straw,dc}^{st+1}+d_{CO_{2},dc}y_{biochar,dc}^{st+1}+e_{CO_{2},dc}y_{straw,dc}^{st+1}+f_{CO_{2},dc}$$

where $\sigma_{see}$, $\sigma_{fer}$, $\sigma_{pes}$, $\sigma_{fue}$, $\sigma_{fil}$, and $\sigma_{lab}$ denote the CO_2_ emission coefficients of seed (kg/kg), fertilizer (kg/kg), pesticide (kg/kg), diesel fuel (kg/kg), agricultural film (kg/kg), and labor force (kg/h) inputs, respectively; $\vartheta_{CO_{2},dc}$ is the CO_2_ emission coefficient; and $a_{CO_{2},dc}$, $b_{CO_{2},dc}$, $c_{CO_{2},dc}$, $d_{CO_{2},dc}$, $e_{CO_{2},dc}$, and $f_{CO_{2},dc}$ are the CO_2_ emission factors for crop c.

The CH_4_ emissions at each stage of agricultural production can be estimated from the cultivated area and the CH_4_ emissions per unit area as follows:

(Equation 34) $$En_{CH_{4},dc}^{st}=A_{area,dc}^{st}{ο}_{c}$$

(Equation 35) $$En_{CH_{4},dc}^{st+1}=\left(1+\vartheta_{CH_{4},dc}\right)En_{CH_{4},dc}^{st}$$

(Equation 36) $$\vartheta_{CH_{4},dc}=a_{CH_{4},c}{\left(y_{biochar,dc}^{st+1}\right)}^{2}+b_{CH_{4},c}{\left(y_{straw,dc}^{st+1}\right)}^{2}+c_{CH_{4},c}y_{biochar,dc}^{st+1}y_{straw,dc}^{st+1}+d_{CH_{4},c}y_{biochar,dc}^{st+1}+e_{CH_{4},c}y_{straw,dc}^{st+1}+f_{CH_{4},c}$$

where ${ο}_{c}$ represents the CH_4_ emissions per unit area (kg/ha); $\vartheta_{CH_{4},dc}$ represents the emission factor for CH_4_; and $a_{CH_{4},c}$, $b_{CH_{4},c}$, $c_{CH_{4},c}$, $d_{CH_{4},c}$, $e_{CH_{4},c}$, and $f_{CH_{4},c}$ represent the CH_4_ emission factors for crop c.

N_2_O emissions attributed to crop N_2_O emissions, fertilizer application and straw return at each stage were calculated as follows:

(Equation 37) $$En_{N_{2}O,dc}^{st}=A_{area,dc}^{st}\left(\nu_{c}+D_{fer,dc}\nu_{fer}\right)$$

(Equation 38) $$En_{N_{2}O,dc}^{st+1}=\left(1+\vartheta_{N_{2}O,c}\right)En_{N_{2}O,dc}^{st}$$

(Equation 39) $$\vartheta_{N_{2}O,dc}=a_{N_{2}O,c}{\left(y_{biochar,dc}^{st+1}\right)}^{2}+b_{N_{2}O,c}{\left(y_{straw,dc}^{st+1}\right)}^{2}+c_{N_{2}O,c}y_{biochar,dc}^{st+1}y_{straw,dc}^{st+1}+d_{N_{2}O,c}y_{biochar,dc}^{st+1}+e_{N_{2}O,c}y_{straw,dc}^{st+1}+f_{N_{2}O,c}$$

where $\nu_{c}$ is the crop N_2_O emission factor (kg/kg); $\nu_{fer}$ is the emission factor of N_2_O from fertilizer (kg/kg); $\vartheta_{N_{2}O,c}$ is the emission factor of N_2_O; and $a_{N_{2}O,c}$, $b_{N_{2}O,c}$, $c_{N_{2}O,c}$, $d_{N_{2}O,c}$, $e_{N_{2}O,c}$ and $f_{N_{2}O,c}$ are the N_2_O emission factors of crop c.

The carbon sequestration effect of agroecosystems is attributed mainly to the application of exogenous substances and the root system of crops in the soil. That is, not only the primary carbon sequestration capacity of crops but also the effects of biochar and straw application on soil organic carbon need to be considered, as shown below:

(Equation 40) $$f_{CS,dc}=f_{CS,dc}^{st}+f_{CS,dc}^{st+1}$$

(Equation 41) $$f_{CS,dc}^{st}=Y_{dc}^{st}C_{c}A_{area,dc}^{st}\left(1-W_{dc}^{st}\right)\left(1+R_{rsr,dc}\right)/H_{c}$$

(Equation 42) $$\begin{array}{l} f_{CS,dc}^{st+1}=(Y_{dc}^{st+1}C_{c}\left(1-W_{dc}^{st+1}\right)\left(1+R_{rsr,dc}\right)/H_{c}+\\ y_{biochar,dc}^{st+1}Cq_{biochar}+y_{straw,dc}^{st+1}Cq_{straw})A_{area,dc}^{st+1} \end{array}$$

where $f_{CS,dc}^{st}$ and $f_{CS,dc}^{st+1}$ represent the carbon sequestration of crop c under treatment d in stages st and st+1, respectively; $W_{dc}^{st}$ and $W_{dc}^{st+1}$ represent the water content of crop c under treatment d in stages st and st+1, respectively; $H_{c}$ represents the economic coefficient of crop c; $R_{rsr,dc}$ represents the root‒crown ratio of the crop; $C_{c}$ represents the carbon uptake rate of crop c; and $Cq_{biochar}$ and $Cq_{straw}$ represent the carbon fixation coefficients of biochar and straw.

###### System’s water footprint function

The water footprint measures the efficiency of water use for agricultural production. If the water footprint intensity is lower, the water use efficiency is greater, and vice versa,^31^ as shown below:

(Equation 43) $$\min\;F_{WF,d}=\sum_{c=1}^{C}f_{CWF,dc}/\left(Y_{dc}^{st}+Y_{dc}^{st+1}\right)$$

where $F_{WF,d}$ is the water footprint intensity under treatment d (m^3^/kg) and $f_{CWF,dc}$ is the water footprint for crop c under treatment d (m^3^/kg).

The water footprint refers to the amount of water required for all products and services consumed in a given period and can be categorized into green water, blue water, and gray water footprints, as shown below:

(Equation 44) $$f_{CWF,dc}=f_{gre,dc}+f_{blu,dc}+f_{gre,dc}$$

where $f_{gre,dc}$ is the green water footprint of crop c under treatment d (m^3^/kg); $f_{blu,dc}$ is the blue water footprint of crop c under treatment d (m^3^/kg); and $f_{gre,dc}$ is the gray water footprint of crop c under treatment d (m^3^/kg).

The green water footprint is the amount of water available for crop growth and is usually expressed in terms of effective rainfall, as shown below:

(Equation 45) $$f_{gre,dc}=\sum_{c=1}^{C}10\left(\sum_{t=1}^{T}P{\left(t\right)}_{dc}^{st}+\sum_{t=1}^{T}P{\left(t\right)}_{dc}^{st+1}\right)/\left(Y_{dc}^{st}+Y_{dc}^{st+1}\right)$$

where P(t) is the effective rainfall (mm) and 10 is the unit conversion factor.

(Equation 46) $$f_{gre,dc}=\sum_{c=1}^{C}10\left(\sum_{t=1}^{T}I{\left(t\right)}_{dc}^{st}+\sum_{t=1}^{T}I{\left(t\right)}_{dc}^{st+1}\right)/\left(Y_{dc}^{st}+Y_{dc}^{st+1}\right)$$

The gray water footprint is the amount of water required to treat pollutants to meet environmental discharge standards, as shown below:

(Equation 47) $$f_{gre,dc}=\sum_{c=1}^{C}\nu_{LRNF}\left(N_{crop,dc}^{st}+N_{crop,dc}^{st+1}\right)/\left(Y_{dc}^{st}+Y_{dc}^{st+1}\right)\left(C_{\max}-C_{n}\right)$$

where $\nu_{LRNF}$ is the leaching rate of nitrogen fertilizer; $C_{\max}$ is the maximum acceptable concentration of nitrogen (mg/L); and $C_{n}$ is the natural concentration of nitrogen (mg/L).

#### Constraints

The synergy of the above objective function is subject to the following conditions:

(1) Electricity supply
  For each treatment, the amount of electricity consumed in the recycling stage to operate the rollers and irrigation pumps should be less than the available electricity, which should be less than the total local electricity supply. The constraints in the recycling stage, including crop biomass power generation, are shown below:
  (Equation 48) $$\sum_{c=1}^{C}A_{area,dc}^{st}C_{ele,dc}^{st}/P_{ele-pur,d}\leq AP_{d}^{st}$$
  (Equation 49) $$AP_{d}^{st}\leq aTAE^{st}$$
  (Equation 50) $$\sum_{c=1}^{C}A_{area,dc}^{st+1}C_{ele,dc}^{st+1}/P_{ele-pue,d}\leq AP_{d}^{st+1}+\sum_{c=1}^{C}\left(Q_{ele-com,dc}^{st+1}+Q_{ele-gas,dc}^{st+1}\right)$$
  (Equation 51) $$AP_{d}^{st+1}\leq aTAE^{st+1}$$
  where $AP_{d}^{st}$ and $AP_{d}^{st+1}$ are the available power supply values (kW.h) for the two stages under treatment d, respectively; $TAE_{d}^{st}$ and $TAE_{d}^{st+1}$ are the total local power supplies (kW.h) for the two stages, respectively; and a is the percentage of irrigation water consumption.
  (Equation 52) $$W{\left(t\right)}_{\min,dc}\leq I{\left(t\right)}_{dc}^{st}\leq W{\left(t\right)}_{\max,dc}$$
  (Equation 53) $$W{\left(t\right)}_{\min,dc}\leq I{\left(t\right)}_{dc}^{st+1}\leq W{\left(t\right)}_{\max,dc}$$
  (Equation 54) $$W{\left(t\right)}_{\min,dc}=10\vartheta_{soil}Z{\left(t\right)}_{r,dc}\theta_{RWP}$$
  (Equation 55) $$W{\left(t\right)}_{\max,dc}=10\vartheta_{soil}Z{\left(t\right)}_{r,dc}\theta_{FC}$$
  where $W{\left(t\right)}_{\min,dc}$ and $W{\left(t\right)}_{\max,dc}$ are the minimum and maximum water contents of crop c (mm), respectively; $Z{\left(t\right)}_{r,dc}$ is the designed depth of the wetted layer of crop c (cm); $\theta_{RWP}$ and $\theta_{FC}$ are the wilting coefficient and field water holding capacity of crop c (cm^3^/cm^3^), respectively; $\vartheta_{soil}$ is the soil capacity (g/kg); and 10 is the unit conversion factor.
(3) Nitrogen supply
  The amount of nitrogen fertilizer used by the crop in the recycling system should be between the maximum and minimum values, with the constraints shown below:
  (Equation 56) $$N_{\min,dc}^{st}\leq N_{dc}^{st}\leq N_{\max,dc}^{st}$$
  (Equation 57) $$N_{\min,dc}^{st+1}\leq N_{dc}^{st+1}\leq N_{\max,dc}^{st+1}$$
  where $N_{\min,dc}^{st}$ and $N_{\max,dc}^{st}$ are the minimum and maximum values of nitrogen fertilizer use for crop c in stage st and $N_{\min,dc}^{st+1}$ and $N_{\min,dc}^{st+1}$ are the minimum and maximum values of nitrogen fertilizer use for crop c in stage st+1, respectively (kg/ha).
(4) Biochar supply
  During the recycling stage, the amount of biochar does not have to exceed the amount of biochar prepared, with the constraints shown below:
  (Equation 58) $$\sum_{c=1}^{C}y_{biochar,dc}^{st+1}\leq \sum_{c=1}^{C}\left(\left(\eta_{gas}Q_{biomass,dc}^{st+1}-\eta_{gas-ele}Q_{ele-gas,dc}^{st+1}\right)+\eta_{pyr}Q_{biomass,dc}^{st+1}\right)$$
(5) Straw return constraints
  The total amount of straw returned to the field does not have to exceed the biomass production of the crop during the recycling stage, with the constraints shown below:
  (Equation 59) $$\sum_{c=1}^{C}y_{straw,dc}^{st+1}\leq \sum_{c=1}^{C}Q_{biomass,dc}^{st+1}$$
(6) Straw allocation weight constraints
  (Equation 60) $$pr_{straw}+pr_{biochar}+pr_{ele}+pr_{sell}=1$$
(7) Nonnegative constraints
  (Equation 61) $$I{\left(t\right)}_{dc}^{st},I{\left(t\right)}_{dc}^{st+1},N_{dc}^{st},N_{dc}^{st+1},y_{biochar,dc}^{st+1},y_{straw,dc}^{st+1},\geq 0$$
  The above model is solved using a fuzzy mathematical approach based on the affiliation function for model solving.

#### Model validation

The coefficient of determination (*R*^*2*^) was used to assess the accuracy of the optimization model. The normalized root mean square error (*nRMS*E) and Willmott consistency index (*WCI*) were combined with R*2* to validate the accuracy of the optimization model. R^2^ and *WCI* values close to 1 indicate good model performance.^23^

(Equation 62) $$R^{2}={\left[\sum_{d=1}^{n}\left(O_{d}-\overset{-}{O}\right)\left(S_{d}-\overset{-}{S}\right)/\sqrt{\sum_{d=1}^{n}{\left(O_{d}-\overset{-}{O}\right)}^{2}{\left(S_{d}-\overset{-}{S}\right)}^{2}}\right]}^{2}$$

(Equation 63) $$nRMSE=100\sqrt{\sum_{d=1}^{n}{\left(S_{d}-O_{d}\right)}^{2}/n}/\overset{-}{O}$$

(Equation 64) $$WCI=1-{\sum_{d=1}^{n}{\left(S_{d}-O_{d}\right)}^{2}/\sum_{d=1}^{n}\left(\left|S_{d}-\overset{-}{O}\right|+\left|O_{d}-\overset{-}{O}\right|\right)}^{2}$$

where n is the test treatment; $S_{d}$ is the simulated value for the recirculation system under treatment d; $O_{d}$ is the observed (experimental) value for the recirculation system under treatment d; and $\overset{-}{S}$ and $\overset{-}{O}$ are the mean values of the simulated and observed values, respectively.

#### Parameter uncertainty and model solution

An integrated management framework requires the input of many social, economic and environmental parameters, which are subjective and uncertain. All the parameters can be obtained through experimental monitoring, by field research or from published references. For example, social parameters may be related to consumer preferences and demand, policy resolutions, and market demand and supply; economic parameters may include crop prices, water costs, and farm purchase prices; and environmental parameters may be related to GHG emissions, soil carbon sequestration, and climate change. When deterministic parameters are input into the model, they tend to limit the range of variation in the model outputs, thus reducing the precision and accuracy of the model. Therefore, to characterize the parameter uncertainties caused by production and human activities more accurately, TIFNs are introduced in this paper.^18^ The use of TIFNs can address challenges related to parameter ambiguity and uncertainty, improve the robustness of the model, and thus produce model output results that are closer to the actual situation and provide more reliable support for decision-making. That is, the accuracy function is used to transform the fuzzy parameters in the input model framework into deterministic parameters, and then the fuzzy planning method is used to transform the multiobjective nonlinear planning model under uncertain conditions into a deterministic single-objective nonlinear planning model to be solved; ultimately, an allocation strategy of water and nitrogen resources that considers a variety of straw utilization scenarios can be generated.

To develop more precise strategies for the green and efficient management of water and fertilizer resources and comprehensively analyze the impacts of extreme precipitation events on water resource use, it is necessary to explore in detail the applicability and possible impacts of various water allocation strategies under different precipitation scenarios. Therefore, precipitation uncertainty can be analyzed through Monte Carlo stochastic simulation.^23^ In this study, the cumulative rainfall for tomato, corn, and soybean crops from seedling to maturity was used in conjunction with Monte Carlo simulation to classify the test years into water-abundant, water-flat, and water-depleted years based on the crop growth characteristics of the study area. Moreover, multiyear rainfall data can objectively reflect local precipitation patterns and provide an important basis for crop growth. Therefore, we used precipitation data from the last 22 years (2000–2022) and calculated corresponding precipitation amounts of 448.1 mm, 320.6 mm, and 271.2 mm on the basis of the P-III curves for precipitation guarantee rates (PGRs) of 25%, 50%, and 75%, respectively. To study the impact of rainfall on irrigation in detail, this study selected the years 2012 (512.6 mm), 2008 (419.5 mm) and 2005 (212.9 mm) to represent abundant, flat and dry water years, respectively, providing a scientific basis for irrigation management.

#### Determination of system quality indicators and straw allocation weights

The indicator evaluation system was constructed according to the hierarchical analysis method (AHP) (Figure 3). Electronic questionnaires were sent to 90 consumers and 10 horticultural experts, and the scoring averages were analyzed to determine the subjective weights of each indicator. Moreover, the entropy weight method was used to calculate the objective weights of the measured values of each indicator. To further improve and optimize the weight allocation process and improve the fidelity and reliability of the model, the game theory combination assignment method was used to determine the indicator weights for the indicator evaluation system (see Table S1).

Different straw allocation weights lead to different water and nitrogen resource allocation scenarios. Hierarchical analysis was used to determine the weights of straw crushed and returned to the field, straw used for biochar preparation, straw used for power generation and straw sold in the recycling stage. The straw recycling scenarios were set up as follows: (a) the four straw reuse scenarios were of equal importance in the recycling stage; (b) the straw return to the field scenario was the most important; (c) the biochar preparation scenario was the most important; (d) the straw power generation scenario was the most important; and (e) the straw sale scenario was the most important. The detailed weights of each allocation scenario are shown in Table 2. To verify the rationality of the weights of the straw allocation scenarios, a consistency test was conducted.^25^ The calculation results show that the consistency ratios of the above weighting scenarios are less than 0.1, satisfying the consistency criteria. Therefore, the devised weights can optimize the inputs of the model and thus assist in generating the best straw allocation scenarios.

### Quantification and statistical analysis

In this study, various statistical analyses and methods were employed to evaluate the performance of the optimization model for sustainable agricultural resource management in a farm ecosystem. The statistical details, including the tests used, sample sizes (n), and definitions of central tendency and dispersion, are primarily documented in the "Results Analysis" section and relevant figure legends. The primary statistical measures used include the coefficient of determination (*R*^*2*^), normalized root mean square error (*nRMSE*), and Willmott's Index of Agreement (*WCI*) to assess the accuracy and reliability of the optimization model. These metrics were applied to compare simulated and observed values for yield, net economic benefit (NEB), total crop quality (TCQ), water footprint (WF), carbon footprint (CF), and biomass. The sample size (n) refers to the number of experimental treatments, which were derived from field trials and model simulations. The central tendency was measured using means, while dispersion and precision were assessed using standard deviations (SD) and standard errors of the mean (SEM), respectively. Confidence intervals were also reported to indicate the reliability of the results. Significance was defined using a P-value threshold of *p*<0.05. The statistical methods used to determine strategies for randomization and stratification were based on the design of the field experiments, which included factorial arrangements of irrigation, nitrogen fertilization, and straw utilization treatments. Sample size estimation was based on the number of replicates in the field trials and the number of scenarios in the model simulations. Data inclusion and exclusion criteria were determined based on the consistency and completeness of the experimental data, with outliers or missing values being excluded from the analysis. To ensure the assumptions of the statistical approach were met, the data were tested for normality and homogeneity of variances. When necessary, transformations were applied to the data to meet these assumptions. The statistical analyses were primarily conducted using software such as Microsoft Excel for basic calculations and data visualization, and specialized software for optimization modeling and Monte Carlo simulations.
