# Supplementary material for: Achieving sustainable goals in agroecosystems through the optimization of agricultural resources integrating the water-nitrogen-carbon nexus
Source: iScience. 2025 Feb 25;28(3):112101. doi: 10.1016/j.isci.2025.112101 (PMC11938145; doi:10.1016/j.isci.2025.112101)
Supplement: Document S1. Figure S1 and Tables S1 and S2 [file mmc1.pdf]

iScience, Volume *28*

Supplemental information

Achieving sustainable goals in agroecosystems through the optimization of agricultural resources integrating the water-nitrogen-carbon nexus

Mo Li, Pingan Zhang, Aizheng Yang, Xiaofang Wang, Yan Sha, and Qiang Fu

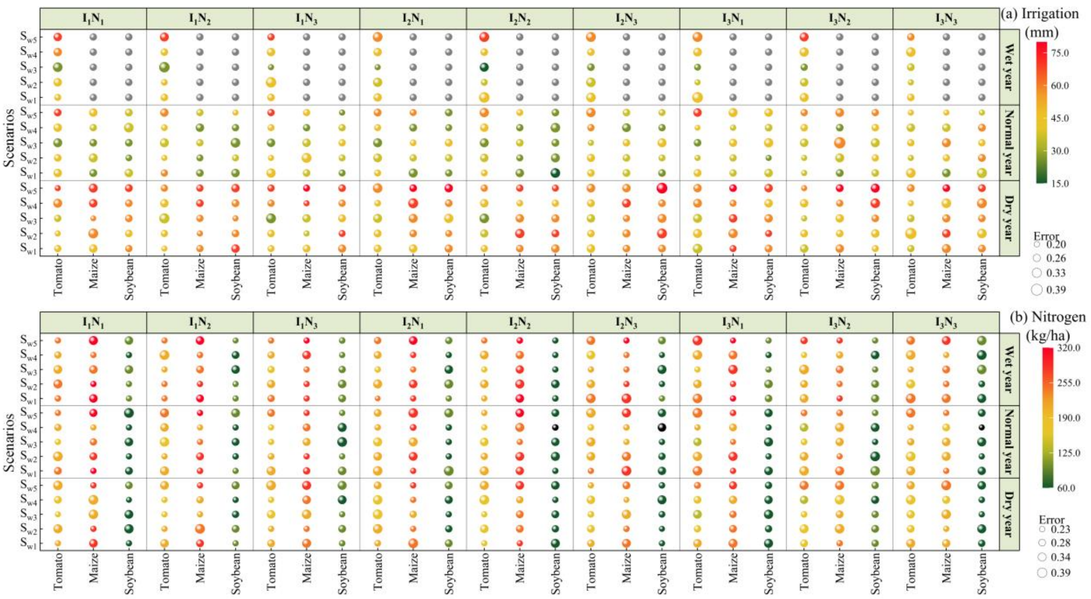

Figure S1 Effect of straw allocation scheme on water and nitrogen resource allocation

Table S1 Indicator weights for evaluation of crop cropping systems

	Methods	OA	SES	VC	SSR	PN	LYP	MCT
Tomato	AHP	0.218	0.129	0.059	0.089	0.277	0.119	0.109
	EWM	0.098	0.179	0.028	0.183	0.249	0.148	0.115
	GCWM	0.104	0.163	0.036	0.175	0.186	0.167	0.168
	Methods	CSE	STC	VC	SES	SSR	PN	
Corn	AHP	0.119	0.185	0.252	0.156	0.119	0.170	
	EWM	0.132	0.088	0.187	0.154	0.209	0.231	
	GCWM	0.125	0.137	0.219	0.155	0.164	0.201	
	Methods	OA	FT	MCT	PN	SSR		
Soybean	AHP	0.242	0.182	0.295	0.121	0.159		
	EWM	0.136	0.288	0.203	0.136	0.237		
	GCWM	0.103	0.316	0.148	0.297	0.135		

Note：AHP is analytic hierarchy process；EWM is entropy weight method；GCWM is game theory combinatorial weighting method；OA is organic acids；SES is soluble solids；VC is vitamin C；SSR is soluble sugar；PN is protein；LYP is lycopene；MCT is moisture content；CSE is cellulose；STC is starch；AAC is amino acid；FT is fat.

Table S2 Results of structural equation modeling of crop cropping systems

Parameter	Relation	Parameter	Standardized Total Effects	Standardized Direct Effects	Standardized Indirect Effects	*P* value
Irrigation	—>	Yield	0.521	0.551	-0.030	**
Irrigation	—>	NEB	0.532	0.532	0	***
Irrigation	—>	TCQ	0.463	0.462	0.001	*
Irrigation	—>	WF	-0.574	-0.623	0.049	*
Irrigation	—>	CF	-0.273	-0.271	-0.002	*
Irrigation	—>	Biomass	0.422	0.422	0	***
Nitrogen	—>	Yield	0.721	0.725	-0.004	***
Nitrogen	—>	NEB	-0.542	-0.546	0.004	**
Nitrogen	—>	TCQ	0.552	0.556	-0.004	***
Nitrogen	—>	WF	-0.114	0	-0.114	0.749
Nitrogen	—>	CF	-0.612	-0.727	0.115	***
Nitrogen	—>	Biomass	0.481	0.516	-0.035	**
Precipitation	—>	Yield	0.183	0.234	-0.051	0.652
Precipitation	—>	Biomass	-0.185	-0.221	0.036	0.564
Straw	—>	Yield	0.181	0.149	0.032	0.642
Straw	—>	NEB	0.522	0.478	0.044	*
Straw	—>	TCQ	0.221	0.326	-0.105	**
Straw	—>	WF	-0.164	-0.224	0.060	0.228
Straw	—>	CF	0.182	0.182	0	*
Biochar	—>	Yield	0.345	0.426	-0.081	*
Biochar	—>	NEB	0.381	0.421	-0.040	*
Biochar	—>	TCQ	0.662	1.236	-0.574	**
Biochar	—>	WF	-0.112	-0.526	0.414	0.964
Biochar	—>	CF	0.572	0.226	0.346	**
